# *Magnetospirillum magneticum* triggers apoptotic pathways in human breast cancer cells

**DOI:** 10.1186/s40170-023-00313-3

**Published:** 2023-08-09

**Authors:** Stefano Menghini, Matej Vizovisek, Jonathas Enders, Simone Schuerle

**Affiliations:** Department of Health Sciences and Technology, Institute for Translational Medicine, ETH Zurich, CH-8092 Zurich, Switzerland

**Keywords:** Magnetotactic bacteria, Cancer therapy, Apoptosis, Cellular proliferation

## Abstract

The use of bacteria in cancer immunotherapy has the potential to bypass many shortcomings of conventional treatments. The ability of anaerobic bacteria to preferentially accumulate and replicate in hypoxic regions of solid tumors, as a consequence of bacterial metabolic needs, is particularly advantageous and key to boosting their immunostimulatory therapeutic actions in situ. While several of these bacterial traits are well-studied, little is known about their competition for nutrients and its effect on cancer cells which could serve as another potent and innate antineoplastic action. Here, we explored the consequences of the iron-scavenging abilities of a particular species of bacteria, *Magnetospirillum magneticum*, which has been studied as a potential new class of bacteria for magnetically targeted bacterial cancer therapy. We investigated their influence in hypoxic regions of solid tumors by studying the consequential metabolic effects exerted on cancer cells. To do so, we established an in vitro co-culture system consisting of the bacterial strain AMB-1 incubated under hypoxic conditions with human breast cancer cells MDA-MB-231. We first quantified the number of viable cells after incubation with magnetotactic bacteria demonstrating a lower rate of cellular proliferation that correlated with increasing bacteria-to-cancer cells ratio. Further experiments showed increasing populations of apoptotic cells when cancer cells were incubated with AMB-1 over a period of 24 h. Analysis of the metabolic effects induced by bacteria suggest an increase in the activation of executioner caspases as well as changes in levels of apoptosis-related proteins. Finally, the level of several human apoptosis-related proteins was investigated, confirming a bacteria-dependent triggering of apoptotic pathways in breast cancer cells. Overall, our findings support that magnetotactic bacteria could act as self-replicating iron-chelating agents and indicate that they interfere with proliferation and lead to increased apoptosis of cancer cells. This bacterial feature could serve as an additional antineoplastic mechanism to reinforce current bacterial cancer therapies.

**Supplementary Information:**

The online version contains supplementary material available at 10.1186/s40170-023-00313-3.

## Background

Cancer is a disease that can arise from various healthy cells in the body displaying continuous and dysregulated proliferation. When tumor cells evade cell cycle control signals and other regulatory cues, they can grow and divide in an uncontrolled manner and eventually spread throughout the body to invade healthy organs and tissues [1]. Recent studies revealed that this crucial cancer hallmark is frequently driven by mutated signaling pathways that compromise cell cycle exit rather than an uncontrolled cell cycle progression [2, 3]. Another main feature of cancer cells is their ability to evade apoptosis, a molecular program executed by activation of caspase signaling, and characteristic for almost all cell types. During apoptosis, healthy cells undergo precisely controlled molecular and physiological changes that lead to inflammation-free cell removal without exerting damage to neighboring cells [4]. Cancer cells, on the other hand, can exploit several ways to avoid the apoptosis program. Amongst these, inhibition of caspases, upregulation of anti-apoptotic BCL-2 proteins, or loss of function of pro-apoptotic proteins such as BAX and BAK are the most prominent [5]. Therefore, it is not surprising that various cancer therapies focus either on preventing uncontrolled cancer proliferation through drugs that force cells to permanently exit the cell cycle or on leveraging the cell’s own death programs to trigger extrinsic or intrinsic apoptotic pathways [6, 7].

Besides conventional cancer treatments such as surgery, chemotherapy, and radiotherapy, highly personalized and more selective approaches such as noncellular and cellular immunotherapies have gained momentum in recent years [8, 9]. As such, bacteria have been increasingly drawing attention in the context of cancer therapy. While the beneficial effects of bacterial infection with respect to tumor regression have already been observed by the American surgeon William Coley in the late nineteenth century [10], it is only because of recent advances in molecular and synthetic biology that the potential of bacteria as living therapeutics is beginning to unfold. Due to their innate capabilities enhanced by genetic engineering techniques, bacteria can exert a number of traits that make them promising candidates in the fight against cancer and other diseases [11, 12]. For example, obligate anaerobes, such as *Clostridium* or *Bifidobacterium*, have been shown to proliferate in no or very low oxygen conditions such as solid tumors [13]. These microorganisms employ their migratory and chemotactic abilities to overcome tissue barriers normally inaccessible for conventional drug-based therapies. To date, a broad number of bacterial classes such as *Clostridium*, *Salmonella*, *Listeria*, *Bifidobacterium*, *Lactobacilli*, *Escherichia*, and *Magnetospirillum* are being increasingly studied for this purpose, with some already advancing to clinical trials [14–16].

Magnetotactic bacteria (MTB) are a group of gram-negative bacteria that are specialized for iron uptake and formation of intracellular, compartmentalized magnetic crystals called magnetosomes [17]. In their natural environment, these facultative anaerobes align along the earth’s magnetic field to reach regions for optimal growth in water or sediments [18, 19]. Additionally, these magnetosomes allow the MTB to be detected and guided by externally applied magnetic fields [20]. In bacterial cancer therapy, their native localization in hypoxic regions of solid tumors can be magnetically increased [15, 21], and their enhanced requirement for iron might cause inducible iron competition and depletion within the tumor environment, possibly resulting in a decreased viability of cancer cells [22].

It is well-known that iron plays a central role in cancer cell metabolism [23–25]. Iron-sulfur groups rely on iron as a cofactor and are essential building blocks present in the active site of many enzymes vital for replication and proliferation, such as DNA polymerase or respiratory complexes [26]. Depleting the tumor environment of iron is thus a recognized therapeutic strategy, commonly referred to as iron chelation therapy, often using naturally derived siderophores extracted from bacteria. These natural iron chelators display a greater affinity for iron than the proteins secreted by eukaryotic cells [27]. While systemic iron depletion can be problematic in cancer patients, siderophores such as deferoxamine (DFO) are currently more commonly used to treat syndromes characterized by excess iron such as hemochromatosis [28, 29]. In a previous study, we analyzed the iron-chelating properties of magnetotactic bacteria AMB-1 and identified a potent siderophore secretion that caused an upregulation of the iron import receptor TfR1 in cancer cells, thus demonstrating iron deprivation [22].

In the present paper, we investigated the correlation between induced iron deprivation and viability of cancer cells in vitro. For this, we first employed AMB-1 bacteria at increasing concentrations and studied their effect on cancer cell proliferation and benchmarked it against fluorouracil (5-FU), a cytotoxic chemotherapeutic drug used to treat cancer. We then determined the number of apoptotic cells when incubating cancer cells with MTB under normoxic and hypoxic conditions. We observed a concentration-dependent activation of executioner caspases as well as caspase-dependent cleavage of PARP-1, thus confirming apoptosis triggering. Finally, an initial screening of distinct human apoptosis-related and stress-related proteins suggested specific alterations when cancer cells were incubated with magnetotactic bacteria. Our findings indicate that the capabilities of *Magnetospirillum magneticum* to reduce cellular proliferation combined with the induction of apoptosis may well serve as an additional strategy to enhance antineoplastic mechanisms in cancer therapy.

## Materials and methods

### Bacterial strain and culture condition

*Magnetospirillum magneticum* AMB-1 (ATCC, Manassas, VA, USA) were first grown anaerobically and then implemented in our cell culture experiments. Bacteria were cultured in liquid medium (ATCC medium: 1653 revised magnetic *Spirillum* growth medium) hereafter referred to as MSGM. MSGM contains per liter 5.0 mL Wolfe’s mineral solution (ATCC, Manassas, VA, USA), 0.45 mL resazurin, 0.68 g of monopotassium phosphate, 0.12 g of sodium nitrate, 0.035 g of ascorbic acid, 0.37 g of tartaric acid, 0.37 g of succinic acid, and 0.05 g sodium acetate. To adjust the pH to 6.75, sodium hydroxide (NaOH) was used. Directly before passaging, MSGM was supplemented with 10 mM ferric quinate (200 ×) and Wolfe’s Vitamin Solution (100 ×) (ATCC, Manassas, VA, USA). Incubation was performed at 30 °C under hypoxic conditions. Passaging was performed weekly, and optical density (OD) measurements were performed using a multiplate reader (Spark, Tecan, Männedorf, Switzerland). The concentration of bacteria was determined via OD measurements by using a calibration curve previously established in the lab (Supplementary file 1, Fig. S1). Bacterial numbers after normoxic and hypoxic co-cultures were assessed, and separate growth curve of AMB-1 was generated additionally (Supplementary file 1, Fig. S2).

### Mammalian cell culture

Human breast cancer cells MDA-MB-231 (ATCC, Manassas, Virginia, USA) were cultured in high glucose Dulbecco’s Modified Eagle’s Medium (DMEM, Invitrogen, Carlsbad, CA, USA) supplemented with 10% fetal bovine serum (FBS, Biowest, Nuaille, France) and 1% penicillin–streptomycin (CellGro, Corning, NY, USA). All cells were incubated at 37 °C in a humidified atmosphere with 5% CO_2_. Cells were passaged twice a week, and they were grown to a confluency of ca. 80% prior to the experiment.

### Co-culture of mammalian cancer cells with magnetotactic bacteria

MDA-MB-231 cells were cultured to a confluency of ca. 80% on either 6-well or 12-well plates, depending on the experiment. The cells were incubated in a humidified incubator at 5% CO_2_ and 37 °C for 24–48 h. Subsequently, the media was changed to FBS supplemented DMEM without penicillin–streptomycin, and different concentrations of *Magnetospirillum magneticum* AMB-1 were added into the wells. Unless specified otherwise, the plates were always placed in a sealable box which was subsequently flushed with nitrogen for 15 min to produce hypoxic conditions. The plates were incubated using this setup at hypoxic conditions at 37 °C for 24–48 h. Using phenol red as pH indicator, the color of the media was monitored throughout the experiment, and a pH in the order of ~ 7.5 was ensured. To benchmark the iron-chelating potential of bacteria against known agents, we used different concentrations (25 µM and 250 µM) of the iron-chelating agent deferoxamine mesylate (D9533, Sigma-Aldrich, St. Louis, MO, USA). To assess the potential of bacteria to induce apoptosis in cancer cells, we used the apoptosis inducer Staurosporine (STS) (LC Laboratories, Woburn, MA, USA) at a concentration of 0.1 µM and the pan-caspase inhibitor Z-VAD-FMK (FMK001, R&D Systems, Minneapolis, MN, USA) at a concentration of 20 µM as control conditions.

### Investigation of cell numbers by Hoechst staining

After the 48-h co-culture experiment, the cells were washed three times with ice-cold Dulbecco’s phosphate-buffered saline solution (DPBS, Gibco, Carlsbad, CA, USA). Cells were subsequentially incubated with 5 µg/mL Hoechst 33342 (H3570, Thermo Fisher Scientific, Waltham, MA, USA) for 15 min before being imaged using a Nikon Eclipse Ti2 microscope (Nikon Instruments, Tokyo, Japan) equipped with a Yokogawa CSU-W1 Confocal Scanner Unit (Yokogawa, Tokyo, Japan) and Hamamatsu C13440-20CU ORCA Flash 4.0 V3 Digital CMOS camera (Hamamatsu photonics, Hamamatsu, Japan). Microscope operation and image acquisition were performed using Nikon NIS-Elements Advanced Research software (Nikon Instruments, Tokyo, Japan). ImageJ2 Version 2.3.0/1.53q (NIH, Bethesda, MD, USA) was used to process the obtained images.

### Investigation of iron content in cellular supernatants

After 24 h in co-culture, the supernatants were removed and analyzed according to manufacturer instructions using a Spectroquant Iron Test (Supelco). Ascorbic acid was additionally added to the medium to reduce all iron ions to Fe(II). This way, the sum of Fe(II) and Fe(III) ions, and thus, the total amount of iron in the media, could be assessed. The resulting complexes in the samples were measured photometrically via absorbance scan using a multiplate reader (Spark, Tecan, Männedorf, Switzerland).

### Proliferation assay

Cell proliferation was assessed using Click-iT Plus EdU Imaging Kit (Thermo Fisher Scientific, Waltham, MA, USA). Proliferation of cancer cells was measured 24 h after incubation with AMB-1 via the incorporation of 4µM 5-ethynyl-2′-deoxyuridine (EdU). Briefly, the cells were fixed with 4% PFA, washed with 3% BSA (PAN-Biotech, Bayern, Germany), and permeabilized with 0.5% Triton-X 100 prior to the addition of 5-ethynyl-2′-deoxyuridine (EdU) at a final concentration of 4 µM. As a positive control, the cytotoxic antimetabolite and thymidylate synthase inhibitor 5-fluorouracil (5-FU) was used at different concentrations (F6627, Sigma-Aldrich, St. Louis, MO, USA). Additionally, the nuclei were stained with 5 µg/mL Hoechst 33342 (H3570, Thermo Fisher Scientific, Waltham, MA, USA). Confocal images were taken, and image acquisition was performed as described above. Image processing was performed using Fiji, and the cells were identified based on their Hoechst labelling by applying Otsu’s auto-threshold clustering algorithm as part of the auto threshold plug-in in Version 1.17.2 [30]. A region of interest (ROI) was determined, and the particles were analyzed by using both size and circularity thresholds. The EdU signal of the cells was then measured as integrated density of the regions of interest obtained in the previous step. An area without any cells was chosen randomly to determine the background signal. Finally, to quantify the EdU signal, the corrected total cell fluorescence (CTCF) was then calculated, and using the total number of cells for each condition, the fraction of EdU-positive cells was determined.

### Apoptosis assay

FITC Annexin V/Dead Cell Apoptosis Kit (Thermo Fisher Scientific, Waltham, MA, USA) was used according to the manufacturer’s instructions to detect early and late apoptotic cell populations after hypoxic and normoxic co-culture experiments. The staining was performed using 1 μL FITC annexin V (solution in 25 mM HEPES, 140 mM NaCl, 1 mM EDTA, pH 7.4, 0.1% bovine serum albumin (BSA) and 1 μL of propidium iodide (PI, 1 mg/mL (1.5 mM) solution in deionized water) in 1 × annexin-binding buffer (50 mM HEPES, 700 mM NaCl, 12.5 mM CaCl_2_, pH 7.4). The supernatants were collected after incubation, and the adherent cells were detached using Enzyme-Free Cell Dissociation Solution Hank’s Based (Merck KGaA, Darmstadt, Germany) and subsequentially added to the supernatants. Different cell populations were quantified with flow cytometry, and the fluorescence emission of 10′000 events was recorded. The analysis was performed at the ETH Zurich FACS Core Facility using a BD LSRFortessa device (BD Biosciences, San Jose, CA, USA) in combination with BD FACSDiva in Version 8.0.1. The obtained data was processed using FlowJo Version 10.6.2 (FlowJo, Ashland, OR, USA). Compensation was performed using single-stained controls (isopropanol-treated cells stained with PI and staurosporine-treated cells stained with FITC annexin V). The gating of the resulting plots was based on an unstained control (Supplementary file 1, Fig. S3).

### Preparation of cell lysates

For preparation of cell lysates, the supernatants were collected following the co-culture experiment, and the cells were detached using Enzyme-Free Cell Dissociation Solution Hank’s Based (1 ×) (Merck KGaA, Darmstadt, Germany) and added to the supernatant. Cells were centrifuged at 800 rcf for 10 min and washed twice with 5mL DPBS. For preparation of the lysates, cell pellets were resuspended in RIPA buffer of pH 8.0 containing 50 mM Tris, 100 mM NaCl, 0.1% w/v SDS, 1% v/v IGEPAL CO-520, 0.5% w/v deoxycholic acid, and 1 mM EDTA. Cells were incubated for 10 min on ice, centrifuged at 13′000 rpm for 15 min, and the soluble lysates were transferred into fresh Eppendorf tubes. Protein concentration was determined using the Pierce BCA Protein Assay Kit (Thermo Fisher Scientific, Waltham, MA, USA) and measuring the absorbance at 562 nm using a multiplate reader (Spark, Tecan, Männedorf, Switzerland).

### Quantification of caspase activity based on cleavage of Ac-DEVD-AFC

Caspase activity was measured with Ac-DEVD-AFC (ENZO Life Sciences AG, Farmingdale, NY, USA) fluorescent substrate. The assay was performed using a buffer containing 100 mM HEPES, 200 mM NaCl, 0.2% (w/v) CHAPS, 20% (w/v) sucrose, 2 mM EDTA, and 20 mM DTT. After addition of the substrate, fluorescence was monitored over 30–60 min (excitation wavelength of 400 nm/emission wavelength of 505 nm) using intervals of 30–60 s. The relative caspase activity for each sample was then calculated as an increase in fluorescence over time.

### Western blotting

Expression and/or activation/processing of apoptosis-related proteins was investigated using Western blot (full blots available in Supplementary file 2, Fig. S12). Equal amounts of total protein (20 µg/lane) were separated on a Bio-Rad Mini-PROTEAN TGX stain-free precast gels 4–20%, and the proteins were blotted onto Bio-Rad Immun-Blot PVDF Membranes. The membranes were blocked in Bio-Rad EveryBlot Blocking Buffer and subsequently incubated with primary antibodies overnight (Supplementary file 1, Table 1). On the next day, the membranes were washed and incubated with the matching secondary antibodies (Supplementary file, Table 1) before Bio-Rad ECL Substrate was applied to capture chemiluminescence with an Azure Biosystems Biomolecular Imager (Azure Biosystems, Dublin, CA, USA).

### Human apoptosis array and human cell stress array for proteome profiling

The levels of human proteins relevant for assessing apoptosis and cell stress were investigated using Human Apoptosis Array Kit (R&D Systems, Minneapolis, MN, USA) and Human Cell Stress Array Kit (R&D Systems, Minneapolis, MN, USA) according to the manufacturer’s protocol. MDA-MB-231 cells were harvested after the co-cultures, and whole-cell lysates were prepared. Briefly, the protein containing lysates were incubated with the provided antibodies spotted assay membranes overnight at 4 °C. On the next day, the membranes were washed repeatedly, and the proteins were detected with streptavidin-HRP and chemiluminescent detection reagents. The resulting signals were then quantified by an Azure Biosystems Biomolecular Imager Sapphire RGBNIR (Azure Biosystems, Dublin, CA, USA) using the imaging software Azure Biosystems Sapphire 1.0.0.1213. The array images were then processed using ImageJ v2.0 (NIH). The signal (integrated pixel density) for each target protein was determined and then normalized to the signal strength of the reference spot. The corresponding signals for each protein were then compared amongst the different membranes (full array analysis are available in Supplementary file 1, Fig. S4 and S5).

### Statistics and data analysis

All graphs and statistical analyses were performed using Prism 8.0 (GraphPad). Statistical significance and number of replicates of the experiments are described in each figure and figure legend. Error bars, where present, indicate the standard error of the mean (SD). *P*-values are categorized as *P < 0.05, **P < 0.01, and ***P < 0.001.

## Results

### Magnetospirillum magneticum AMB-1 affects cancer cell numbers in vitro and leads to reduced cellular proliferation of MDA-MB-231 cells

Hypoxia is an important mechanistic aspect involved in tumorigenesis and one of the characteristic features of the tumor microenvironment [31]. To closely mimic this property of solid tumors in our in vitro experiments, we used a chamber to replicate a low-oxygen environment [22] and thus enable us to study the interplay between bacteria and cancer cells in conditions resembling those normally found in hypoxic regions of solid tumors (Supplementary file 1, Fig. S6).

**Fig. 1:**
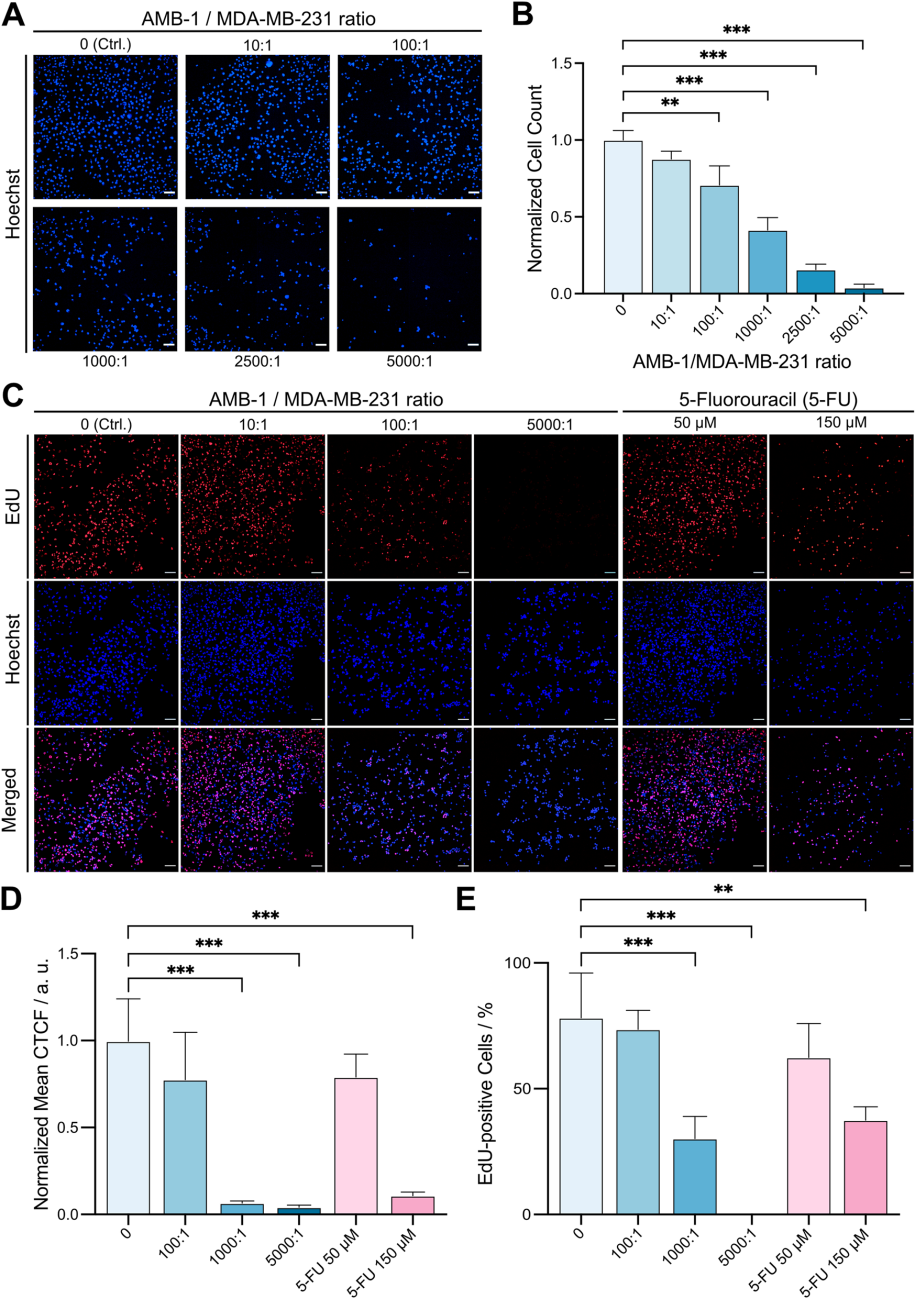
Quantification of cell numbers and determination of de novo DNA synthesis in MDA-MB-231 cells incubated with AMB-1. **A **Representative images of human breast cancer cells co-cultured under hypoxic conditions for 48 h with different ratios of AMB-1 bacteria. Images show MDA-MB-231 cells stained with Hoechst 33342 (blue), scale bar: 100 µm. **B **Graphical representation of the fluorescence intensities measured in Fig. 1A. Cell counts were normalized to the control condition (*n* = 3 biological replicates per condition, statistical significance was assessed with an ordinary one-way ANOVA test). **C **EdUKit was used to examine cellular proliferation after 24-h exposure to AMB-1 and 5-FU (positive control). MDA-MB-231 cells were marked by EdU (red) and Hoechst 33342 (blue), scale bar: 100 µm. **D **The EdU fluorescence intensity of the images in Fig. 1C was assessed, and the normalized mean CTCF was plotted (*n* = 3 biological replicates per condition, statistical significance was assessed with a one-way ANOVA test). **E** The extent of EdU-positive cells was determined by dividing EdU-stained cells by the number of Hoechst 33342 stained cells (*n* = 3 biological replicates per condition, statistical significance was assessed with an ordinary one-way ANOVA test)

First, we studied the behavior of breast cancer cells upon exposure to live AMB-1 in an environment with a reduced presence of oxygen. Accordingly, MDA-MB-231 cells were inoculated with bacteria and then incubated for 48 h before confocal fluorescent microscopy was performed to characterize the co-cultures. This analysis showed a drastic drop in the number of cells in the wells with rising concentrations of bacteria (Fig. 1A). Moreover, the observed effect was concentration dependent, and a statistically significant decrease of cancer cells could be detected even at AMB-1:MDA-MB-231 ratios as low as 100:1 (10^7^ AMB-1) (Fig. 1B). Similar outcomes could also be detected when co-cultures were performed under normoxic conditions (Supplementary file 1, Fig. S7), albeit slightly stronger. This can be explained by higher bacterial proliferation under normoxic condition [32, 33] which impacts cell adherence.

We were thus interested in investigating metabolic effects induced by the presence of AMB-1 on cancer cells and potential links to their strong iron sequestering capabilities which could induce disturbances of iron homeostasis. We had previously determined the potency of AMB-1 to deplete iron and found that AMB-1 produced siderophores equivalent to a concentration of 3.78 µM ± 0.117 µM deferoxamine (DFO) [22]. To further assess iron sequestration by AMB-1, we quantified the iron level in medium after the co-culture experiments and then investigated whether the proliferation of human cancer cells is impacted by the bacteria. For this and all further investigations, a 24-h timepoint was chosen. After hypoxic co-culture experiments, the supernatant was collected, and the amount of iron was examined. The concentration of iron was significantly lower when AMB-1 were added to the cell culture, suggesting a positive correlation between iron consumption and the presence of bacteria in the cell culture media (Supplementary file 1, Fig. S8). After confirming the decreased level of this vital nutrient in the media, the potential antiproliferative effect of bacteria on breast cancer cells was investigated. DNA de novo synthesis was measured through incorporation of a fluorescent thymine analog (EdU) in proliferating cells. The fluorescent images showed a clear decrease of EdU fluorescence suggesting that proliferation of cancer cells decreases when incubating them with increasing number of bacteria. As a positive control and to benchmark the measured levels of proliferation decrease, the cytotoxic chemotherapeutic drug 5-fluorouracil (5-FU) was used. We observed a similar antiproliferative effect at a concentration of 150 µM (Fig. 1C), and the fluorescence of EdU-positive cells was then measured, normalized to the control condition, and plotted (Fig. 1D/E). The bacteria showed a significant impact on cellular proliferation once the ratio of bacteria to cells reached a value of 1000:1 or higher. This drastic decrease of EdU incorporation in breast cancer cells could analogously be observed when incubating the cells with 5-FU (150 µM). Additionally, we measured the integrated density of EdU signal and normalized this value by the number of viable cells (marked with Hoechst). Similarly, the fraction of EdU-positive cells in the population correlated with the trends observed in the previous results. By applying Otsu’s auto-threshold clustering algorithm, only 37.7% of the cancer cells incorporated EdU when treated with 5-FU (150 µM). The proliferative cell population decreased even further when incubating them with 1 × 10^8^ (1000:1) bacteria (30.4%) while reaching values close to 0% when inoculating with 5 × 10^8^ bacteria (5000:1). The obtained data sets could be further validated through an additional positive control using PFA for cell fixation prior to incubation, and in this case, no EdU incorporation could be detected (Supplementary file 1, Fig. S9).

These experiments demonstrated that AMB-1 affect cancer cell proliferation and thus directly impact the number of cancer cells that is reached in a low oxygen in vitro setup over a 48-h incubation period. Moreover, the de novo synthesis of DNA was hampered when MDA-MB-231 cells were exposed to living *Magnetospirillum magneticum*, suggesting that bacteria impact cancer cell proliferation in a concentration-dependent manner.

### Increasing number of AMB-1 in co-culture elicits apoptosis in MDA-MB-231 cancer cells

**Fig. 2:**
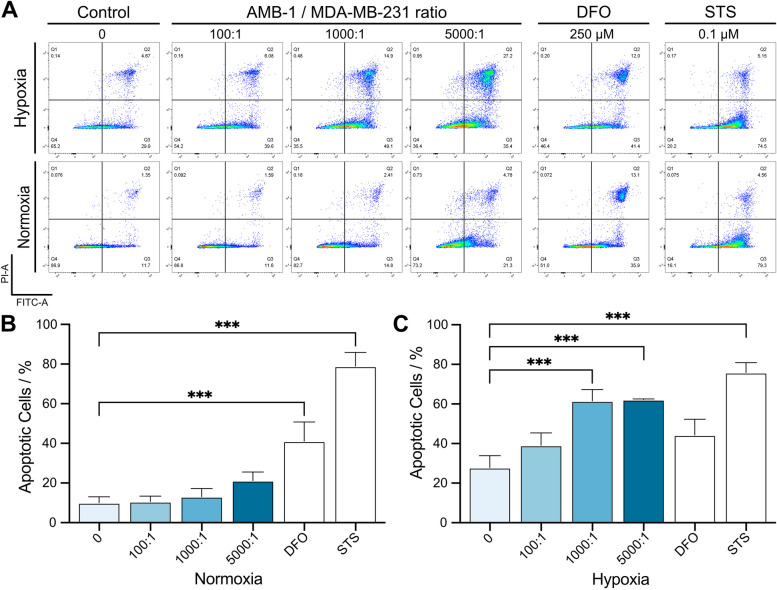
Quantification of early and late apoptotic cell populations of breast cancer cells exposed to AMB-1 bacteria. **A **Representative flow cytometry plots displaying early and late apoptotic cells, deferoxamine (DFO), and staurosporine (STS). X-axis and Y-axis represent Annexin V (FITC-A) and propidium iodide (PI-A), respectively. **B** and **C** Late (Q2) and early apoptotic cells (Q3) were summed and plotted as the total percentage of apoptotic cells. MDA-MB-231 cells were exposed to various conditions and cultured in either a normoxic (**B**) or a hypoxic (**C**) environment (*n *= 3 biological replicates per condition (*n* = 2 for STS); statistical significance was assessed with an ordinary one-way ANOVA test)

Evasion, impairment, or inhibition of apoptotic signaling pathways is a mechanism that can lead to the development and spread of malignant tumors. It is therefore not surprising that promoting pro-apoptotic signaling remains one of the fundamental approaches in cancer therapy. With the following experiments, we aimed to investigate whether *Magnetospirillum magneticum* can affect cancer cell metabolism not only by hampering proliferation but also by influencing the cancer cells towards triggering apoptosis. To determine the size of apoptotic populations, breast cancer cells were stained with annexin V-FITC and PI after 24 h of co-cultures with magnetotactic bacteria under normoxic or hypoxic conditions. A flow cytometry analysis was performed to determine the populations of living (double negatives), early apoptotic (Annexin V positive/PI negative), or late apoptotic cells (double positives) (Fig. 2A). This analysis demonstrated that co-cultures under normoxic conditions resulted in a moderate increase in apoptotic cell populations when MTB were inoculated at rising concentrations. However, it is only by incubating MDA-MB-231 with either DFO, a strong iron chelator, or Staurosporine (STS), a highly potent inductor of apoptosis, that a substantial increase of apoptotic cells could be detected. Accordingly, DFO and STS increased the fraction of apoptotic cells by 31.1% and 68.9% compared to the control condition (Fig. 2B). Interestingly, the facultatively anaerobic microaerophilic bacteria AMB-1 exerted a completely different effect on breast cancer cells in tumor-like microenvironment with a reduced level of oxygen. While the Annexin V-positive cells, following exposure to DFO and STS, closely correlate with the values reported for the normoxic conditions (+ / − 3%), living AMB-1 had a substantially stronger effect under hypoxic conditions and increased the fraction of apoptotic MDA-MB-231 cells. Already at lower ratios of bacteria-to-cancer cells, a substantial increase in the size of apoptotic cell populations could be measured in comparison to the control (+ 11.2%). When the ratio was further increased to 1000:1 and 5000:1, the fraction of apoptotic cell population reached + 33.6% and + 34.3%, respectively (Fig. 2C). The tumor-like hypoxic environment recreated in vitro seemed to significantly impact the ability of living AMB-1 to induce apoptosis in MDA-MB-231 cells. Furthermore, the implementation of this environmental condition seemed to affect the apoptotic rate of breast cancer cells even without the addition of external reagents as cells appeared to be more prone to cell death when subjected to sub-optimal growth conditions like oxygen deprivation (Supplementary file 1, Fig. S10).

Overall, living and proliferating magnetotactic bacteria can induce death of breast cancer cells, which is strongly dependent on the amount of oxygen that is present in the environment.

### Activation of caspases and detection of apoptotic markers upon exposure to AMB-1

Apoptosis or programmed cell death in mammalian cells is executed by caspases, cysteine proteases that cleave their substrate proteins specifically after aspartates. During apoptosis, caspases cleave many different structural and functional cell proteins, thereby giving apoptosis its distinct biochemical and morphological features [34]. Once pro-apoptotic signals are transmitted to the target cells, caspases undergo a series of structural changes that lead to their activation and execution of the apoptosis program. Therefore, detection of caspase activity together with caspase substrates is a reliable molecular marker to assess the extent of apoptosis [35].

**Fig. 3:**
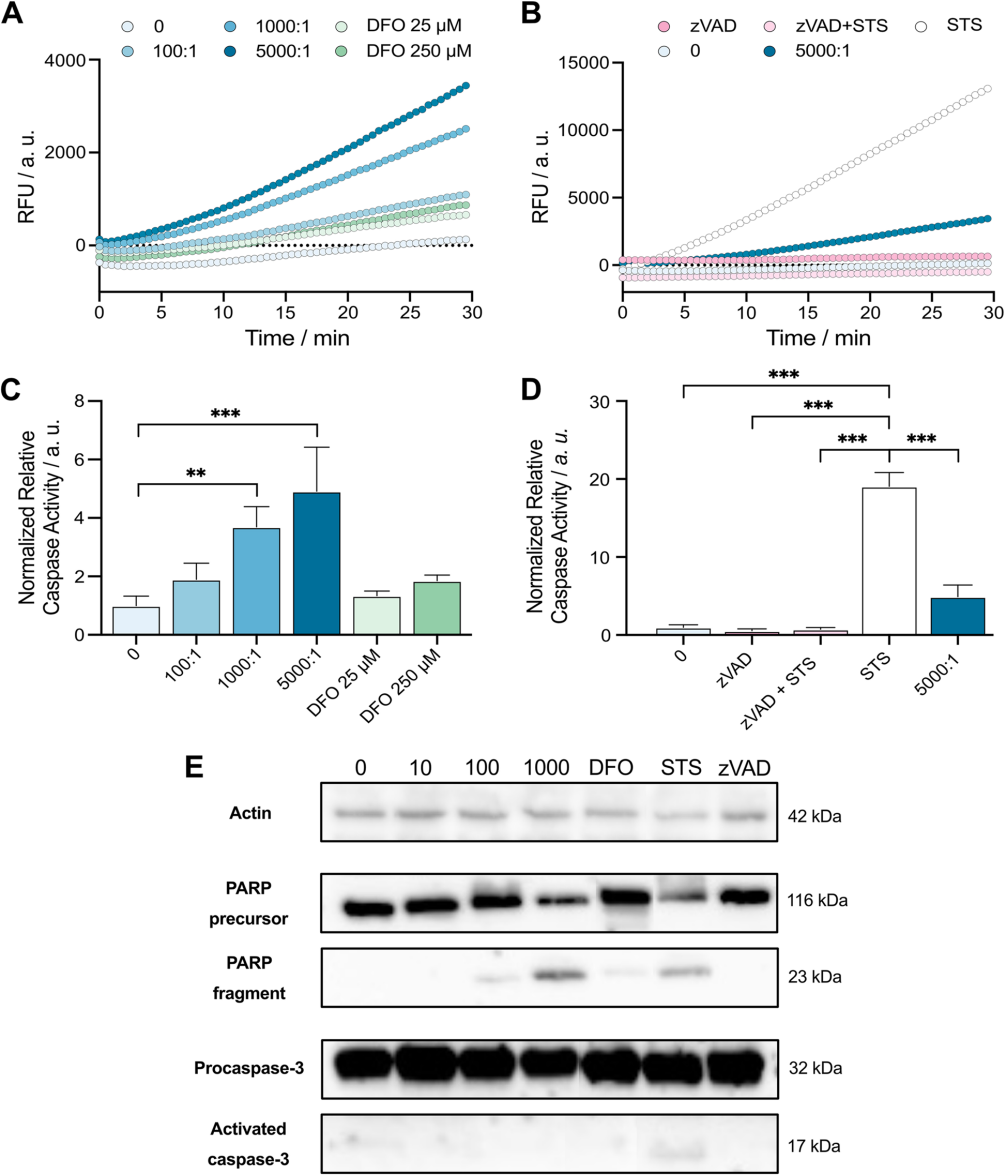
Measurement of caspase activity and identification of apoptotic markers induced by AMB-1 exposure. **A** and **B **Caspase activity measured over 30 min. The curves display the impact on breast cancer cells caused by different concentrations of AMB-1 bacteria and DFO (**A**) and the relative positive and negative controls, STS and zVAD (**B**). **C** and **D **Relative caspase activity extrapolated from graphs (**A**, **B**) and normalized to the control condition (*n* = 4 biological replicates per condition, statistical significance was assessed with an ordinary one-way ANOVA test). **E **Western blot analysis of breast cancer cell lysates. MDA-MB-231 were treated for 24 h in a hypoxic environment with either AMB-1, DFO, STS, and zVAD. Actin was used as a loading control for the analysis of the levels of protein detected. Full images of the membranes can be found in the Supplementary file 2 (Fig. S12)

To investigate the extent of apoptotic changes and confirm the triggering of apoptosis in cancer cells co-cultured with bacteria, the activity of executioner caspases was measured with the fluorescent caspase substrate Ac-DEVD-AFC, which enables relative quantification of caspase activity. In this assay, endogenous caspase activity was determined based on the velocity of the cleavage reaction, which is a reliable indicator of the extent of apoptotic caspase activation, regardless of whether apoptosis was triggered via intrinsic or extrinsic pathway. We observed that both the low (25 µM) and the high (250 µM) concentration of the iron chelator DFO had a very subtle effect on caspase activity. On the other hand, AMB-1 showed a steady increase in activity with an increasing count of the bacteria (Fig. 3A). This effect could be observed even at very low AMB-1:MDA-MB-231 ratios, i.e., as low as 100:1 (1.9-fold increase). A statistically significant activation of executioner caspase was also detected when the bacteria-to-cancer cell ratio was increased to 1000:1 and 5000:1, with a 3.7-fold and 4.9-fold increase in caspase activity (Fig. 3B). Unsurprisingly, the potent apoptosis-inducer STS showed the highest cleavage rate of substrate over time indicating a high activation of executioner caspases (Fig. 3C). In fact, incubation of the breast cancer cells with STS as positive control resulted in a 19.1-fold higher level of caspase activity in comparison to the control condition (Fig. 3D). Additionally, a pan-caspase inhibitor Z-VAD-FMK (zVAD) was implemented in the assay to prevent the triggering of apoptosis and thus confirm that apoptosis was triggered via a caspase-dependent mechanism (Fig. 3C, D). To confirm that the detected signal caused by bacteria was the consequence of activated caspases, an additional incubation of AMB-1 with the inhibitor zVAD was performed, showing a substantial decrease in activity (Supplementary file 1, Fig. S11). A similar trend could be observed when the inhibitor was incubated with either the supernatants of bacterial cultures or catechol siderophores (3,4-dihydroxybenzoic acid) serving as control conditions, supporting that not the bacteria themselves, but rather their secretions, are responsible for caspase activation (Supplementary file 1, Fig. S11).

Next, we performed an immunologic detection of apoptotic markers with Western blot. PARP-1 is a marker for apoptosis triggering because it is one of the substrates cleaved by executioner caspases during the course of the apoptotic program. To evaluate the extent of apoptosis, we thus focused on detecting PARP-1 caspase cleavage product with a molecular weight of approximately 23 kDa. While the strength of the full-length PARP-1 band was comparable amongst all the conditions except for 1000:1 (AMB-1:MDA-MB-231 ratio) and STS, we detected a strong band for PARP-1 cleaved fragment in these two conditions indicating that executioner caspases had to be active (Fig. 3E). Also, in this case, we could observe that the effects depend on the intensity of apoptotic stimulus, and a faint band was visible also when incubating the cells with bacteria at a ratio of 100:1 and with the iron chelator DFO. While STS and high concentrations of AMB-1 resulted in PARP-1 cleavage suggesting apoptosis triggering, 100:1 and DFO seem to have triggered apoptosis as well, even though at a lower extent (Fig. 3E). We further followed the activation of executioner caspase-3 from the proenzyme to mature caspase-3 using Western blot. While no apparent difference could be detected at the level of caspase-3 precursor, we observed a faint band corresponding to the size of the mature caspase-3 p17 small subunit in case of STS (Fig. 3E).

Altogether, these findings show that the bacterial strain AMB-1 can trigger apoptosis in breast cancer cells but to lesser extent than prominent apoptosis inducers, like staurosporine.

### Magnetotactic bacteria can affect the cancer cell protein levels

**Fig. 4:**
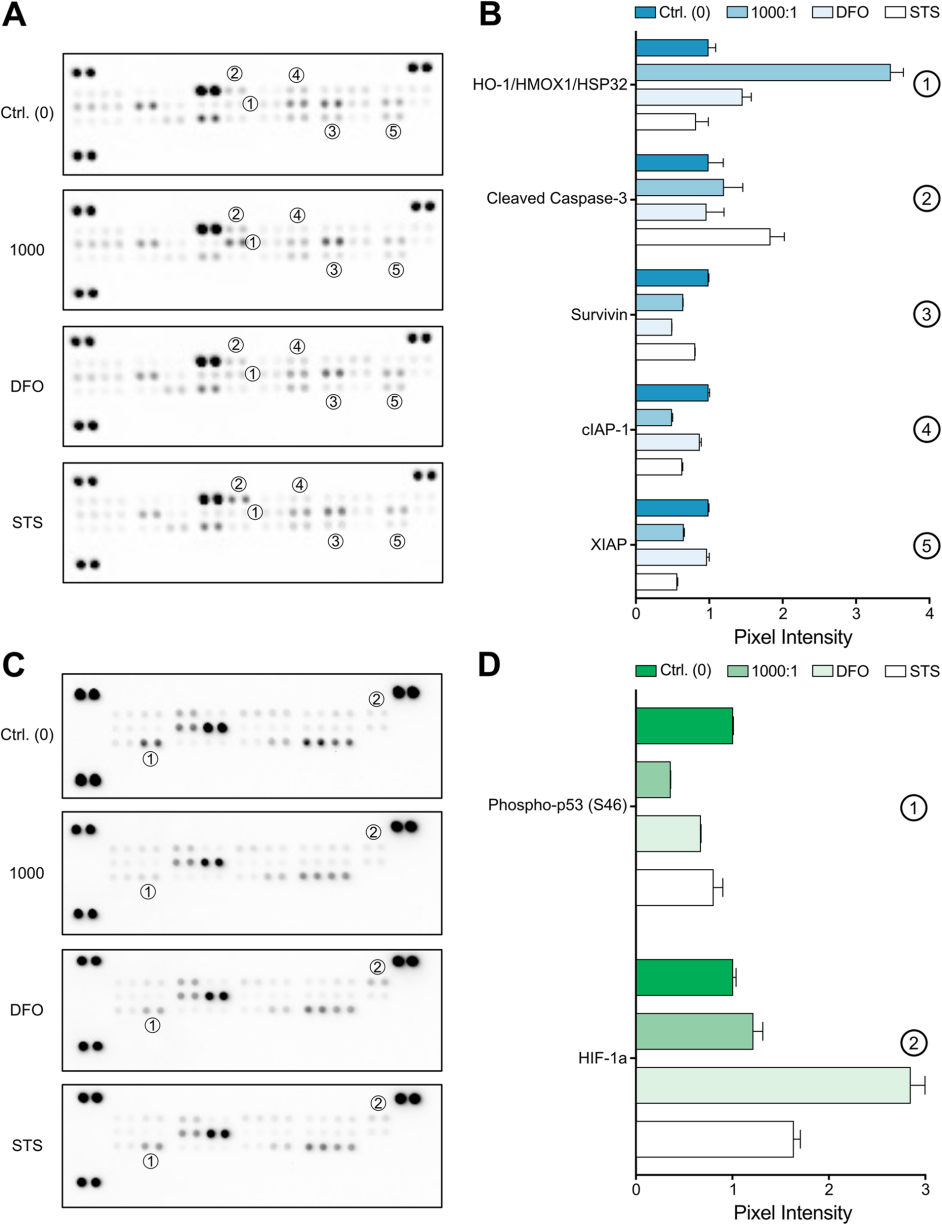
Alteration of protein levels in human breast cancer cells incubated with AMB-1. **A **Human apoptosis array analysis of MDA-MB-231 cells treated with AMB-1 (1000:1), STS, and DFO. **B **Protein expression of HO-1/HMOX1/HSP32, cleaved Caspase-3, Survivin, cIAP-1, and XIAP were found to be altered at a higher extent in at least one of the tested conditions. The protein expressions were normalized to the control and plotted (mean ± SD of duplicates on array). **C **Human stress array analysis of MDA-MB-231 cells treated with AMB-1 (1000:1), STS, and DFO. **D **Protein expression of Phospho-p53 (S46) and HIF-1a was altered with a fold change larger than 1.5 compared to the control condition. The two protein expressions were normalized to the control and plotted (mean ± SD of duplicates on array)

To determine the effects of AMB-1 and compare them to other stimuli that can lead to apoptosis, an initial screen of the levels of apoptosis-related proteins was performed with protein-antibody arrays to generate a more global picture of how MDA-MB-231 cells are impacted by bacteria. For this, cancer cells were incubated for 24 h with either 10^8^ AMB-1 bacteria (1000:1 ratio), 0.1 µM STS, or 250 µM DFO. The resulting lysates were used to detect the levels of 35 different proteins directly or indirectly involved in apoptosis. The signal intensities of the target protein spots on the membranes were analyzed, and relative changes in several apoptosis-related protein levels could be detected between samples (Fig. 4A). Interestingly, HO-1/HMOX1/HSP32, a protein shown to be involved in iron homeostasis and ferroptosis [36, 37], seemed to be highly upregulated (3.6-fold) when the cells were co-cultured with AMB-1 bacteria and slightly upregulated (1.5-fold) when the cells were treated with DFO (Fig. 4A). In agreement with the previous experiments, the activation of caspase-3 was observed and a 1.9-fold increase in intensity of the active caspase-3 form was detected when the cancer cells were incubated with STS. Furthermore, the anti-apoptotic protein Survivin was lower in MDA-MB-231 cells that were exposed to either AMB-1 or DFO. Lastly, AMB-1 and STS seemed to have a similar effect on the levels of the caspase inhibitors XIAP and C-IAP decreasing them by more than 1.5-fold versus the untreated control condition. These results suggest that AMB-1 bacteria could potentially sensitize breast cancer cells towards apoptosis not only by inducing the expression of pro-apoptotic proteins but also by decreasing the levels of anti-apoptotic proteins.

To verify that the apoptosis induced in MDA-MB-231 cells by AMB-1 was not simply linked to stress caused by the sheer number of bacteria, a stress array assay was utilized to detect relative changes of several stress-related protein markers. This initial screen indicated that most of these proteins were found unperturbed by the investigated treatments, and only the expression levels of phospho-p53 (S46) and HIF-1a were altered (> 1.5-fold) (Fig. 4B). Accordingly, MDA-MB-231 exposed to 10^8^ live AMB-1 bacteria displayed a 2.9-fold drop in phospho-p53 (S46) of 2.9-fold versus the control condition. The treatment of cells with 250 µM DFO on the other hand led to an increase of HIF-1a protein expression  by 2.9-fold in comparison to the untreated sample (Fig. 4B).

Taken together, these results suggest the active involvement of living *Magnetospirillum magneticum* in directing cancer cells towards apoptosis while having negligible effects on the level of cellular stress.

## Discussion

Bacteria fulfil many criteria that an ideal anticancer therapeutic should have. It is therefore not surprising that their suitability and significance as a novel tool for cancer therapy are becoming increasingly recognized [38]. Current cancer therapies face many challenges in terms of on-site delivery, therapeutic efficiency, and system toxicity that are often difficult to overcome using conventional therapeutic designs. Moreover, the hypoxic environment in tumors represents another substantial barrier for conventional anticancer therapies since it has been shown that hypoxic solid tumors often exhibit a reduced sensitivity to therapeutic agents [39]. These particular conditions are favorable for growth of obligate and facultative microorganisms, leading to a preferential and selective infiltration within the solid tumors, thus reinforcing their use as new chemotherapeutics in future cancer management strategies [40].

In this study, we attempted to recreate similar conditions as present in the hypoxic regions of solid tumors in vitro and investigated the effect of *Magnetospirillum magneticum*, a unique strain of facultative anaerobic bacteria, on breast cancer cells MDA-MB-231. The magneto-aerotaxis properties of this strain are advantageous not only for reaching regions of oxygen concentration for optimal growth [41, 42] but also because their elevated need for iron could be leveraged to deprive cancer cells of this vital nutrient [22]. We first investigated what part of the original living and adherent cancer cell population could be identified after a 48-h hypoxic incubation with different ratios of AMB-1. Unsurprisingly, the number of breast cancer cells (marked by Hoechst) dropped significantly with increasing numbers of living and proliferating bacteria, suggesting that they had a strong impact on cancer cells and their adherence. We hypothesized that the iron depletion capacity of AMB-1, which we here reconfirmed, was linked to the observed results via potential disturbances in iron homeostasis. To further investigate the possible resulting effects on cellular proliferation, a thymine analog (EdU) was added to the cultures, while the chemotherapeutic agent 5-FU was used as a positive control. The antimetabolite drug 5-FU inhibition of thymidylate synthase results in an arrest of cells in the G1 phase of cell cycle, corresponding to an inhibition of cell proliferation [43, 44]. This comparison revealed that AMB-1 bacteria elicited a robust antiproliferative effect culminating with a nearly complete arrest of proliferation when the ratio of bacteria-to-cancer cells was increased to 5000:1. Incubation of cancer cells with 10^8^ bacteria (1000:1 condition) resulted in a percentage of EdU-positive cells that was very similar to the 5-FU-positive control, suggesting a successful inhibition of proliferation. When the concentration of bacteria was increased even further (5000:1), the percentage of proliferating cancer cells dropped almost to 0%, thus confirming that AMB-1 bacteria can cause cell cycle arrest in cancer cells. These findings suggest that AMB-1 exert antiproliferative effects, which are likely driven by the metabolic disturbance (possibly through iron chelation) [22] resulting in an inhibition of de novo DNA synthesis in MDA-MB-231 cells.

Once we determined that the population of living cells decreases, and that proliferation was hampered by AMB-1, we investigated whether the observed increase in dying MDA-MB-231 was a consequence of apoptosis. For this, we determined the extent of apoptotic populations under normoxic (21% O_2_, 5% CO_2_) and hypoxic conditions. Magnetosome biosynthesis has been reported to require anoxic or microaerobic conditions which correlates with an increased iron uptake by MTB [45]. Apoptotic cues induced by iron deprivation would therefore be expected to be greater at lower oxygen concentrations. For this experiment, the iron chelator DFO and the potent apoptosis inducer staurosporine were utilized as controls, as it has been shown before that they can induce apoptotic changes in cancer cells [46, 47]. As expected, both agents elicited a substantial increment in the population of apoptotic cells in comparison to non-treated controls. Although only a very slight increase in apoptotic cells could be noted when inoculating AMB-1 with cancer cells under normoxic conditions, this effect increased by several folds when the co-culturing was performed under hypoxic conditions. The fraction of apoptotic cells upon incubation with 10^8^ (1000:1) and 5 × 10^8^ (5000:1) AMB-1 was larger than the ones observed upon DFO treatment and essentially reached similar extents of dying/dead cells as observed with STS. Thus, a hypoxic environment seems to be essential not only for the viability of AMB-1 but also for the observed killing of breast cancer cells. While the effect of DFO and STS was proven to be potent but mostly unaffected by the levels of oxygen in cell culture, an incubation of MDA-MB-231 with ratios of 100:1, 1000:1, and 5000:1 AMB-1 resulted in a significant difference when applying a hypoxic in vitro setup. Thus, hypoxia emerged to be necessary for any pro-apoptotic effects elicited by AMB-1, which is in line with the known growth requirements of *Magnetospirillum magneticum*, that proliferate and thrive in low oxygen conditions [20, 42].

After confirming that exposure of breast cancer cells to living AMB-1 resulted in increased cancer cell death, we further screened the involvement of apoptotic caspases in a fluorometric cleavage assay with Ac-DEVD-AFC. Measuring the cleavage rate of this substrate reflects the extent of activation of executioner caspases-3, -6, and -7, which is an established hallmark of apoptosis and can thus serve as a confirmation that apoptosis was triggered [48, 49]. Unsurprisingly, incubation of cancer cells with STS resulted in a fast and strong cleavage of Ac-DEVD-AFC, confirming that STS-induced apoptosis resulted in the activation of caspases [50]. On the other hand, DFO showed little to no activation of caspases under hypoxic conditions. In case of AMB-1, we observed an increasing activation of endogenous caspasesthat correlated with the number of bacteria added to the cultures. Although the highest concentration of bacteria (5 × 10^8^) did not reach the extent of caspase activation observed in case of STS, bacterial presence resulted in 4.9-fold higher relative apoptotic caspase activity than in the control. Furthermore, the decrease in extent of measured activity when the caspase inhibitor zVAD was added to co-cultures with either AMB-1 bacteria or their supernatant suggested that the detected signal was indeed a consequence of activated caspases (Supplementary file 1, Fig. S11).

To further investigate these observations and confirm apoptosis triggering, we studied the caspase-dependent proteolytic processing of PARP-1 during our co-culture experiment as a potent marker of apoptosis. Poly(ADP-ribose) polymerase is a DNA-repair enzyme with important roles in many forms of cell death [51], and proteolytic cleavage of PARP-1 by caspases is a hallmark of apoptosis [52]. We monitored the molecular state of PARP-1 via Western blot and investigated whether proteolytic processing and characteristic PARP-1 cleavage fragments indicative of apoptosis could be observed when bacteria and cancer cells were co-cultured. Reporter PARP-1 fragments were observed at 23 kDa, which subsequently increased in intensity with a rising number of AMB-1, therefore, suggesting a ratio-dependent extent of activation of apoptotic caspases. Similarly, DFO could induce PARP-1 proteolytic cleavage, although to a much lower extent, when compared to STS treatment and the highest number of AMB-1. In addition, we also investigated the activation of caspase-3, one of the crucial mediators of apoptosis, known to be involved in the cleavage of PARP-1 and several other key proteins that give apoptosis its distinct biochemical and morphological features [53]. Here, we observed a band at 17 kDa, corresponding to the cleaved caspase-3, clearly noticeable in case of STS, which was associated with the highest levels of caspase activity. Collectively, these findings suggest that AMB-1 not only have a role in triggering of apoptosis and the associated caspase activation but also lead to generation of typical apoptosis markers such as the proteolytic fragment of PARP-1.

To get a more integral picture of how apoptotic markers are impacted in breast cancer cells upon different stimuli, we used a protein antibody array to simultaneously investigate the relative levels of 35 apoptosis-related proteins. This experiment revealed an upregulation of HO-1/HMOX1/HSP32 by more than 3.5-fold versus the control when cells were exposed to living bacteria. Although its role is not yet fully understood, studies have reported a correlation of HO-1/HMOX1/HSP32 with enhanced cell death in many cancers [54]. Additionally, there are reports of a fundamental role of HO-1 in ferroptosis [36, 55] and of enhanced HO-1 activity when cells seek to increase the level of cellular iron and promotion of ferritin production [37]. Therefore, it is likely that AMB-1 induces apoptosis by interfering with the iron metabolism machinery of breast cancer cells. A slight increase in HO-1/HMOX1/HSP32 expression observed in case of DFO strengthens the hypothesis that the perturbation of the iron metabolism is a key mechanism that leads to the observed death of MDA-MB-231 cells. Additionally, we again detected an elevated activation of caspase-3 (processing of procaspase-3 to mature caspase-3) for the STS condition and to a minor extent for the 1000:1 condition. This correlates with the findings of the Western blot analysis and the caspase activity assays where this crucial apoptotic regulator [53] was indeed activated at higher levels. Interestingly, both DFO and 10^8^ AMB-1 (1000:1) caused a downregulation of the caspase inhibitor Survivin, possibly suggesting a correlation between the level of iron and its expression. Survivin, together with other IAPs, plays an important role in eliminating apoptotic caspase activity by interfering with their activation and thus preventing apoptosis triggering and is often highly expressed in cancers [56]. Its decrease upon exposure to AMB-1 shows that these bacteria could impact cellular levels of both pro-apoptotic as well as anti-apoptotic proteins. In fact, two additional anti-apoptotic proteins, X-linked inhibitors of apoptosis (XIAP) and cellular inhibitors of apoptosis (cIAP), were found to be decreased after cancer cells were incubated with AMB-1 or STS. These two IAP family members prevent the execution of apoptosis either by inhibiting caspases (XIAP) or by repressing the triggering of extrinsic apoptotic pathway (cIAP) [57].

Finally, to confirm that the observed effects of bacteria on cancer cells were due to their involvement in perturbing the iron metabolism rather than elevated stress levels caused by the presence of bacteria, a screen of 26 different human stress-related proteins was performed. This assay revealed only very minor changes at the protein level. Only two proteins displayed changes higher than 1.5-fold, namely the hypoxia-inducible factor 1a (HIF-1a) and the Phospho-p53 (S46). Even though not at great extents, the increase in HIF-1a expression was mainly detected when incubating the breast cancer cells with DFO. DFO-triggered apoptosis is coupled to activation of HIF-1a [58, 59] which supports the current observations. However, under hypoxic conditions, HIF-1a is already actively involved in apoptosis [60], which might be the reason for the less pronounced apoptotic effects of DFO under hypoxia (Supplementary file 1, Fig. S10). The lack of augmented HIF-1a protein levels for the AMB-1 condition, on the other hand, might suggest that magnetotactic bacteria do elicit alternative apoptotic pathways. Besides HIF-1a, Serine 46 (S46) phosphorylation on p53 was found to be higher in apoptotic cancer cells [61, 62]. Nonetheless, all of our treatments seemed to induce a downregulation of this protein compared to controls, and especially when exposed to AMB-1, the cancer cells generally displayed a downregulation of Phospho-p53 (S46). Although no mechanistic conclusion can be made about this observation, it might be linked to the genetic background of the MDA-MB-231 cells which have increased levels of a mutant p53 [63].

The performed arrays uncovered several proteins displaying altered levels, therefore allowing for the first assumptions to be made regarding the pathways involved in apoptotic cues caused by AMB-1 bacteria. Nonetheless, further elucidation and targeted validation of the proteins of interest would be required to ascertain which mechanisms are altered during the induction of apoptosis by AMB-1.

## Conclusion

Bacteria have many features that open an opportunity space for their future use as anticancer therapeutics. Amongst advantageous properties of bacteria in this context are their motility, which allows them to move towards the site where treatment is needed, their ability to carry a payload of drug molecules, and for anaerobic and facultative anerobic strains, the increased accumulation in the hypoxic regions of the tumors. This study focused on *Magnetospirillum magneticum*, a strain of magnetotactic bacteria with iron-chelating capabilities and the preference of growing and thriving in hypoxic environments. With a demonstrated ability to successfully impact the metabolism of breast cancer cells in vitro, AMB-1 were shown to affect the proliferation of MDA-MB-231 cells and trigger apoptosis. Previous studies highlighted the ability of magnetotactic bacteria to infiltrate across biological barriers in vitro and in vivo and to proliferate in solid tumors [15]. With this work, we demonstrate the capability of AMB-1 to induce apoptosis in cancer cells possibly through iron competition in the tumor microenvironment [22]. The innate capability of *Magnetospirillum magneticum* to activate apoptosis in cancer cells thus represents a promising strategy to develop new tools for cancer treatment, which could, especially when successfully integrated with the existing chemotherapeutic drugs, substantially enhance the current therapeutic strategies in the field of bacterial cancer therapy.

### Supplementary Information

**Additional file 1: Supplementary file 1.** **Fig. S1.** Calibration curve representing the OD values plotted against the number of magnetotactic bacteria. Equation: y = 1.333E-09x + 6.926E-02, R-squared value: R² = 0.9994. **Fig. S2.** Assessment of bacterial growth over time via cell count and OD600 measurement. Different bacteria to cell ratios were counted after 0 h, 24 h, and 48 h when co-cultured in either (A) hypoxic or (B) normoxic conditions (*n*=3 biological replicates). (C) Growth of bacteria in MSGM was measured over 8 days by regularly taking OD measurements (*n*=3 biological replicates). **Fig. S3.** Gating strategy for the assessment of apoptotic cell populations measured by flow cytometry. **Fig. S4.** Full human apoptosis array analysis of MDA-MB-231 cells treated with AMB-1 (1000:1), STS and DFO. **Fig. S5.** Full human stress array analysis of MDA-MB-231 cells treated with AMB-1 (1000:1), STS and DFO. **Fig. S6.** Comparison of in vitro cancer cell cultures under either hypoxic or normoxic conditions. (A) Representative fluorescence and brightfield images after 2 h, 24 h, and 48 h of MDA-MB-231 cells stained with Image-IT Green Hypoxia Reagent (green), (scale bar: 50 µm). (B) Picture of the custom-made hypoxia box with an inlet and an outlet to allow for nitrogen flushing. **Fig. S7.** Quantification of adherent cells after incubation with AMB-1 (A) Representative images of human breast cancer cells co-cultured under normoxic conditions for 48 h with increasing ratios of AMB-1 bacteria. Images show MDA-MB-231 cells stained with Hoechst 33342 (blue), scale bar: 100 µm (B) Graphical representation of the fluorescence intensities measured in A. Cell counts where normalized to the control condition (*n*=3 biological replicates per condition, statistical significance was assessed with an ordinary one-way ANOVA test). **Fig. S8.** Determination of iron levels in the medium after hypoxic co-cultures (A) Spectroquant Iron Test was used to investigate the extent of iron consumption over 24 h. Cancer cells were either left untreated (0 Ctrl.) or incubated with AMB-1 at a bacteria to cell ratio of 1000:1. An absorbance scan was performed and the conditions were compared to the control (cell culture medium) (B) The intensities measured at wavelengths between 480-530 nm were averaged and the resulting mean values were normalized to the control condition and plotted as a column chart (*n*=3 biological replicates per condition, statistical significance was assessed with an ordinary one-way ANOVA test). **Fig. S9.** Determination of de novo DNA synthesis in MDA-MB-231 (control conditions). (A) EdU Kit was used to examine cellular proliferation after 24 h. Cells were either left untreated (0 Ctrl.) or treated with 4 % PFA as a negative control for cellular proliferation. Unstained cells were used as a further control. MDA-MB-231 cells were marked by EdU (red) and Hoechst 33342 (blue), scale bar: 100 µm. (B) The EdU fluorescence intensity of the images in Figure S2 was assessed and the normalized mean CTCF was plotted (*n*=3 biological replicates per condition, statistical significance was assessed with a one-way ANOVA test). (C) The extent of EdU-positive cells was determined by dividing EdU-stained cells by the number of Hoechst 33342 stained cells (*n*=3 biological replicates per condition, statistical significance was assessed with an ordinary one-way ANOVA test). **Fig. S10.** Quantification of early and late apoptotic cell populations of breast cancer cells incubated with several conditions in either a hypoxic or normoxic environment. *n*=3 biological replicates per condition (*n*=2 for STS), statistical significance was assessed with an ordinary one-way ANOVA test. **Fig. S11.** Measurement of caspase activity over 60 minutes. Mean activity of executioner caspases, with and without inhibition using zVAD, was measured (*n*=2 biological replicates per condition). The curves display the impact on MDA-MB-231 cells caused by (A) AMB-1 bacteria (1000:1), (B) Supernatant of bacteria cultured in DMEM, (C) Supernatant of bacteria cultured in MSGM, and (D) Catechol siderophores (1µM). (E) Relative change of caspase activity over time extrapolated from graphs A, B, C, and D was normalized to the condition with lowest activity change (1000:1 + zVAD). (F) Inhibitory effect of zVAD for each condition represented as the mean fold decrease of activity (*n*=2 biological replicates per condition, statistical significance was assessed with an ordinary one-way ANOVA test). **Table 1.** List of antibodies used for Western Blotting.

**Additional file 2: Supplementary file 2.** **Fig. S12.** Western blot analysis of breast cancer cell lysates. MDA-MB-231 were treated for 24 h in a hypoxic environment with either AMB-1, DFO, STS or, zVAD. Full membranes scans of PARP (A), Caspase 3 (B), and Actin (C) are hereby presented. Marked in red are lanes belonging to additional conditions that were not further investigated.

## Data Availability

All data analyzed during this study are included in this published article and its supplementary information files. The associated raw data are available from the corresponding author on reasonable request.

## Abbreviations

BCA: Bicinchoninic acid
BSA: Bovine serum albumin
CHAPS: 3-[(3-Cholamidopropyl) dimethylammonio]-1-propanesulfonate
CTCF: Corrected total cell fluorescence
DMSO: Dimethyl sulfoxide
DPBS: Dulbecco’s phosphate-buffered saline
DFO: Deferoxamine
DMEM: Dulbecco’s Modified Eagle’s medium
EdU: 5-Ethynyl-2′-deoxyuridin
EDTA: Ethylenediaminetetraacetic acid
FITC: Fluorescein isothiocyanate
HEPES: 4-(2-Hydroxyethyl)-1-piperazineethanesulfonic acid
MTB: Magnetotactic bacteria
PFA: Paraformaldehyde
PI: Propidium Iodide
RIPA: Radioimmunoprecipitation assay
TfR1: Transferrin receptor
STS: Staurosporine
zVAD: Pan-caspase inhibitor Z-VAD-FMK
5-FU: 5-Fluorouracil
