# Supplementary material for: Magnetospirillum magneticum triggers apoptotic pathways in human breast cancer cells
Source: Cancer Metab. 2023 Aug 9;11:12. doi: 10.1186/s40170-023-00313-3 (PMC10410830; doi:10.1186/s40170-023-00313-3)
Supplement: Supplementary file 1 — Additional file 1: Supplementary file 1. Fig. S1. Calibration curve representing the OD values plotted against the number of magnetotactic bacteria. Equation: y = 1.333E-09x + 6.926E-02, R-squared value: R² = 0.9994. Fig. S2. Assessment of bacterial growth over time via cell count and OD600 measurement. Different bacteria to cell ratios were counted after 0 h, 24 h, and 48 h when co-cultured in either (A) hypoxic or (B) normoxic conditions (n=3 biological replicates). (C) Growth of bacteria in MSGM was measured over 8 days by regularly taking OD measurements (n=3 biological replicates). Fig. S3. Gating strategy for the assessment of apoptotic cell populations measured by flow cytometry. Fig. S4. Full human apoptosis array analysis of MDA-MB-231 cells treated with AMB-1 (1000:1), STS and DFO. Fig. S5. Full human stress array analysis of MDA-MB-231 cells treated with AMB-1 (1000:1), STS and DFO. Fig. S6. Comparison of in vitro cancer cell cultures under either hypoxic or normoxic conditions. (A) Representative fluorescence and brightfield images after 2 h, 24 h, and 48 h of MDA-MB-231 cells stained with Image-IT Green Hypoxia Reagent (green), (scale bar: 50 µm). (B) Picture of the custom-made hypoxia box with an inlet and an outlet to allow for nitrogen flushing. Fig. S7. Quantification of adherent cells after incubation with AMB-1 (A) Representative images of human breast cancer cells co-cultured under normoxic conditions for 48 h with increasing ratios of AMB-1 bacteria. Images show MDA-MB-231 cells stained with Hoechst 33342 (blue), scale bar: 100 µm (B) Graphical representation of the fluorescence intensities measured in A. Cell counts where normalized to the control condition (n=3 biological replicates per condition, statistical significance was assessed with an ordinary one-way ANOVA test). Fig. S8. Determination of iron levels in the medium after hypoxic co-cultures (A) Spectroquant Iron Test was used to investigate the extent of iron consumption over 24 h. Can [file 40170_2023_313_MOESM1_ESM.docx]

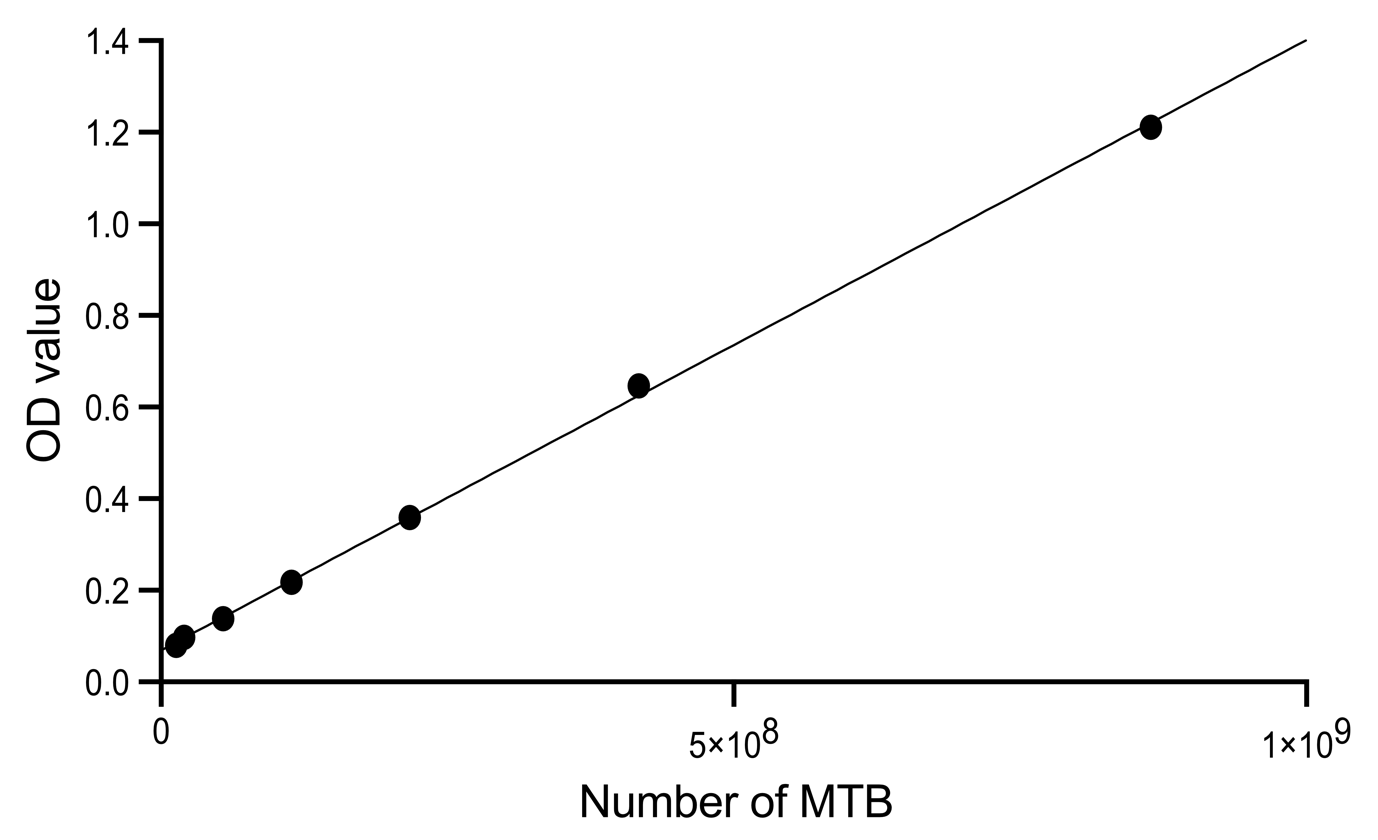


**Figure S1:** Calibration curve representing the OD values plotted against the number of magnetotactic bacteria. Equation: y = 1.333E-09x + 6.926E-02, R-squared value: R² = 0.9994.

**Figure S2:** Assessment of bacterial growth over time via cell count and OD600 measurement. Different bacteria to cell ratios were counted after 0 h, 24 h, and 48 h when co-cultured in either **(A)** hypoxic or **(B)** normoxic conditions (n=3 biological replicates)**.** **(C)** Growth of bacteria in MSGM was measured over 8 days by regularly taking OD measurements (n=3 biological replicates)**.**


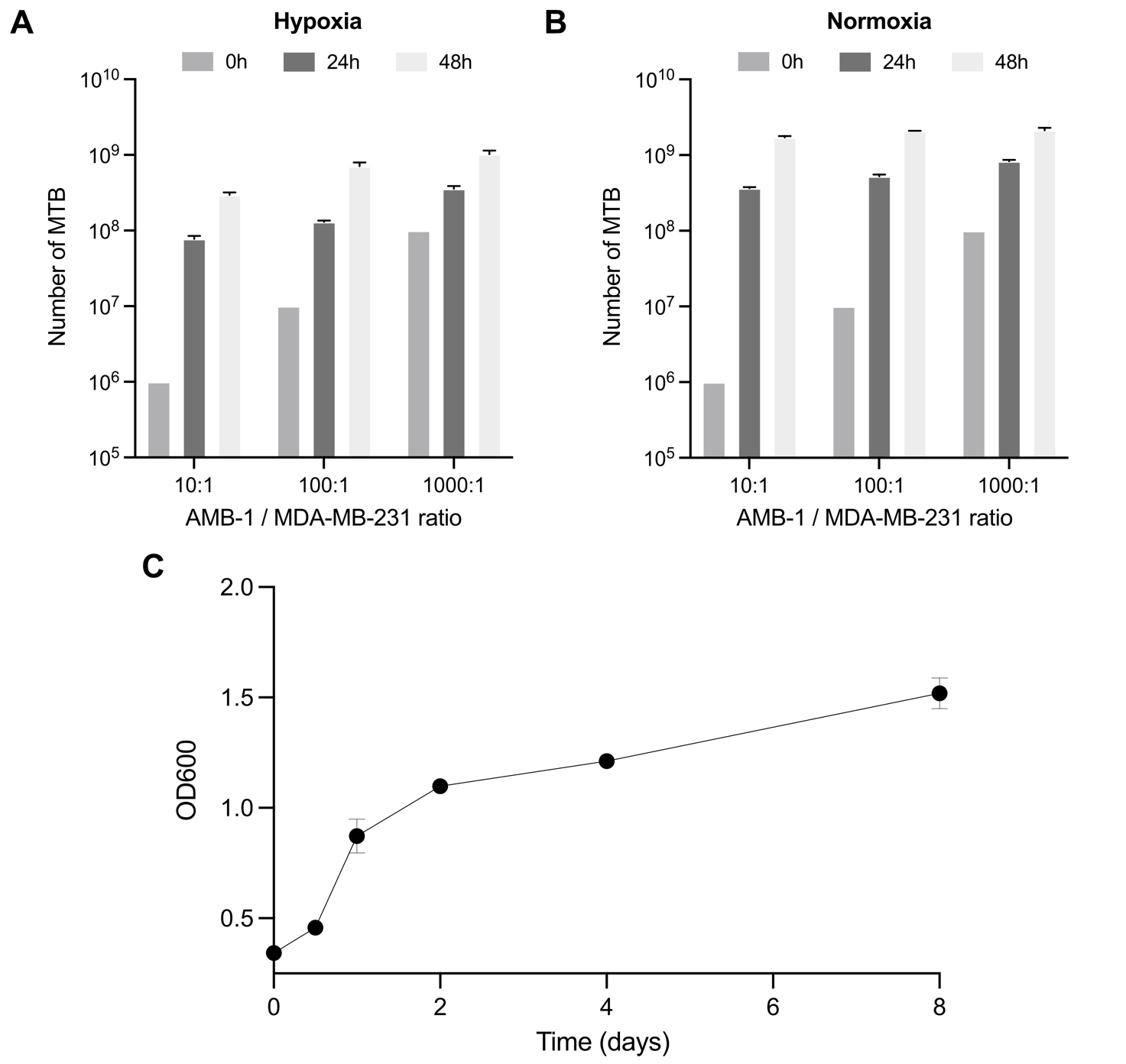


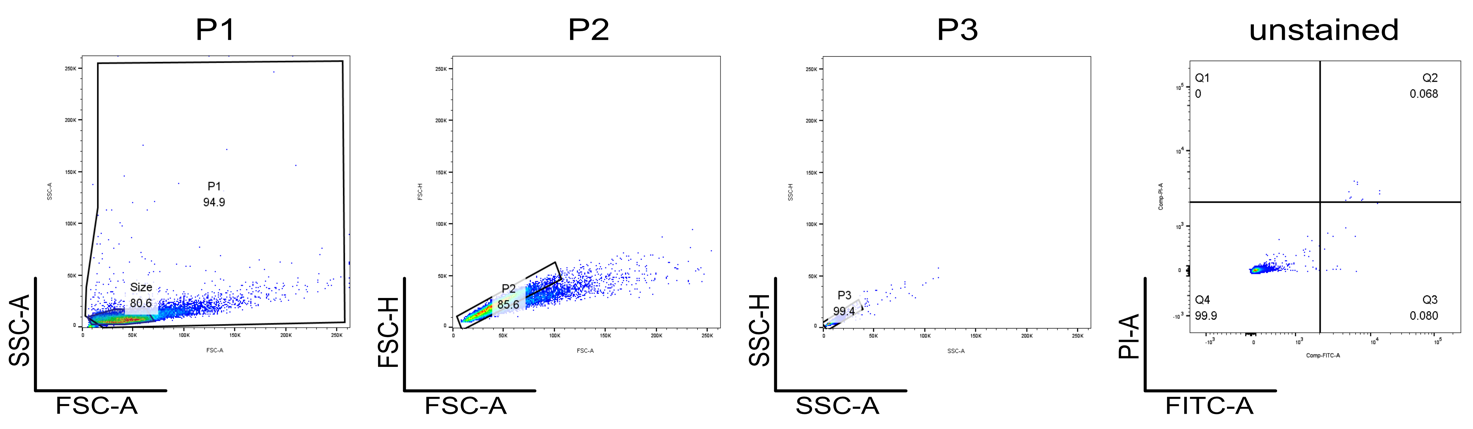


**Figure S3:** Gating strategy for the assessment of apoptotic cell populations measured by flow cytometry.


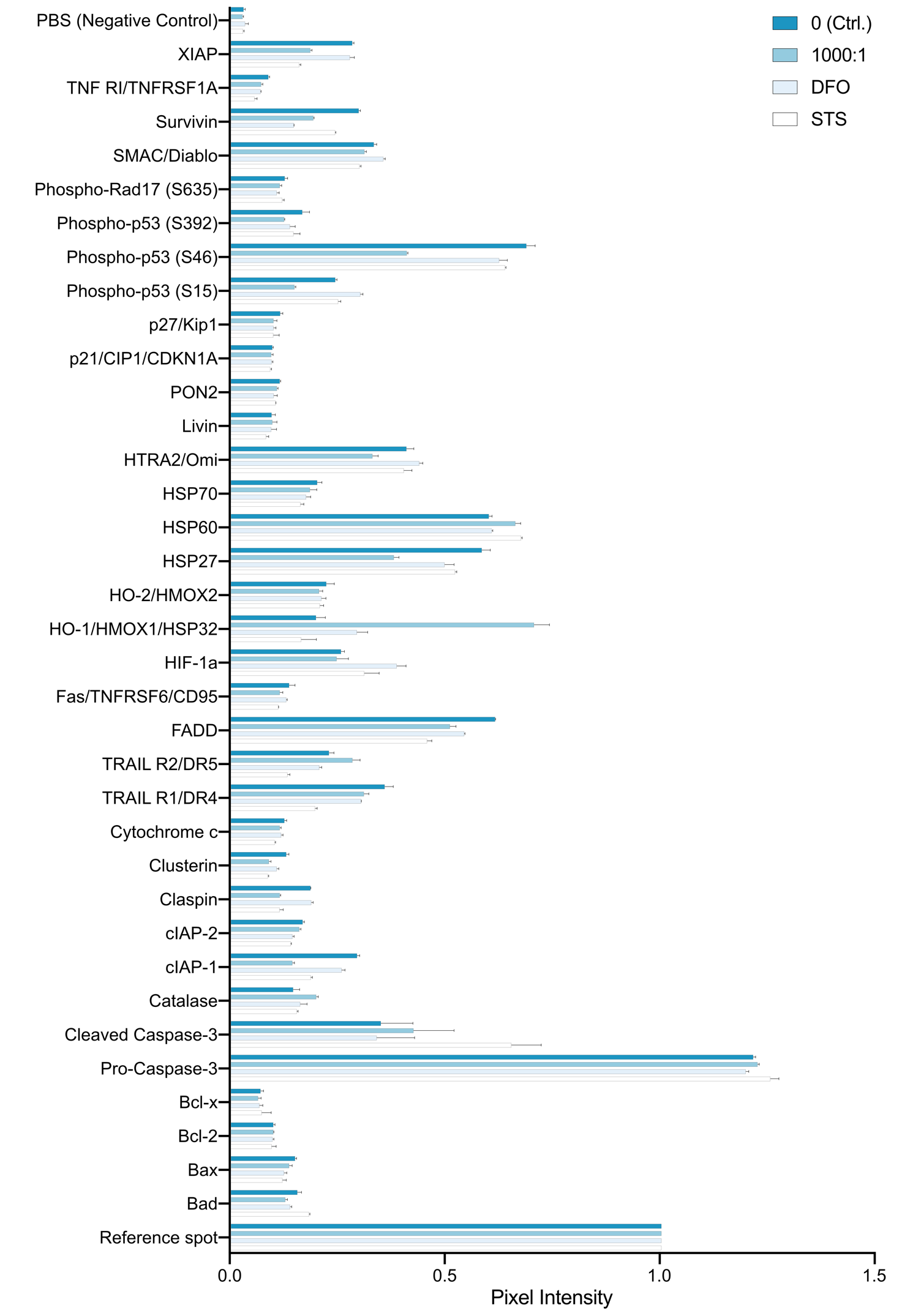


**Figure S4:** Full human apoptosis array analysis of MDA-MB-231 cells treated with AMB-1 (1000:1), STS and DFO.


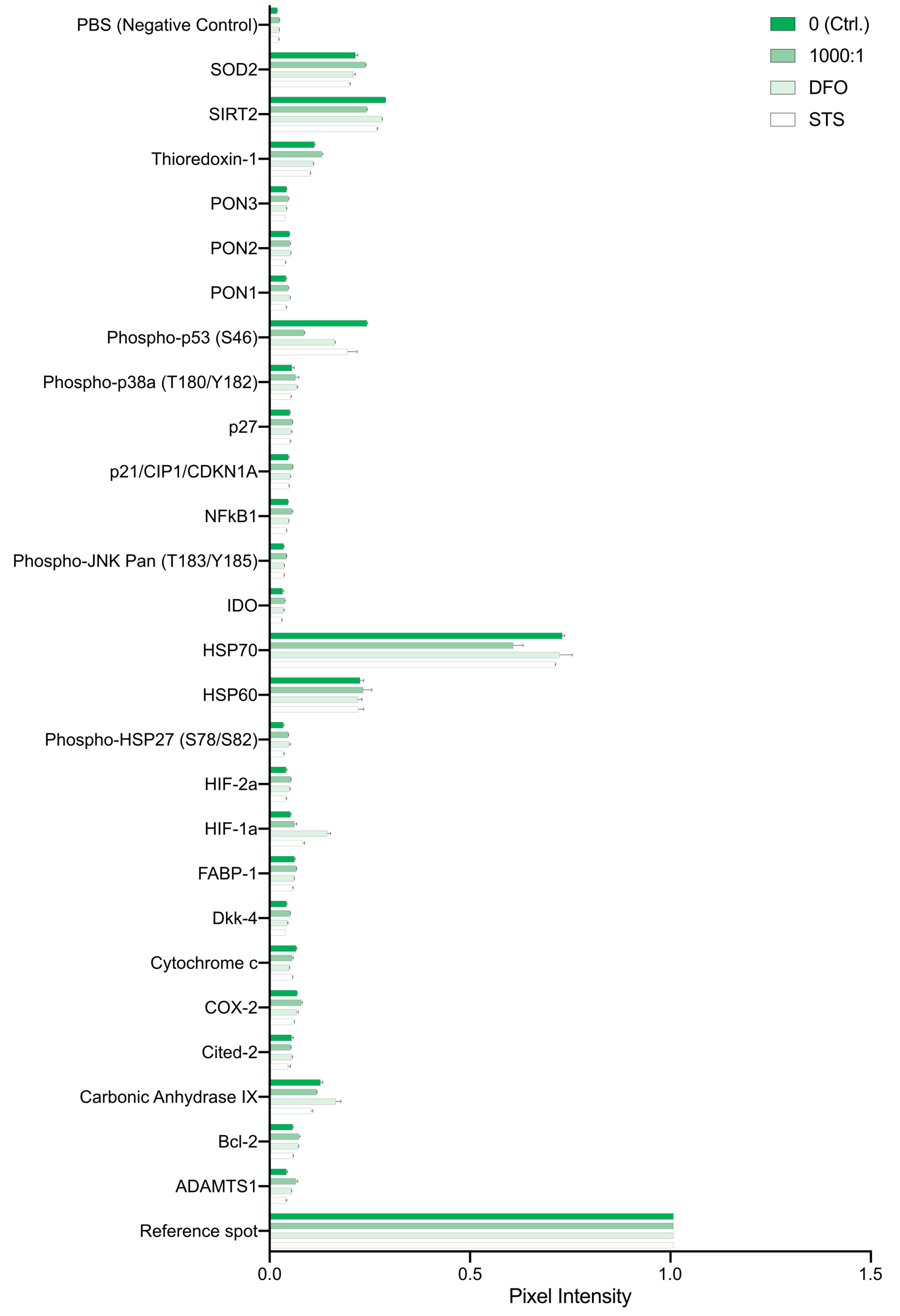


**Figure S5:** Full human stress array analysis of MDA-MB-231 cells treated with AMB-1 (1000:1), STS and DFO.

**Figure S6:** Comparison of in vitro cancer cell cultures under either hypoxic or normoxic conditions. **(A)** Representative fluorescence and brightfield images after 2 h, 24 h, and 48 h of MDA-MB-231 cells stained with Image-IT Green Hypoxia Reagent (green), (scale bar: 50 µm). **(B)** Picture of the custom-made hypoxia box with an inlet and an outlet to allow for nitrogen flushing.


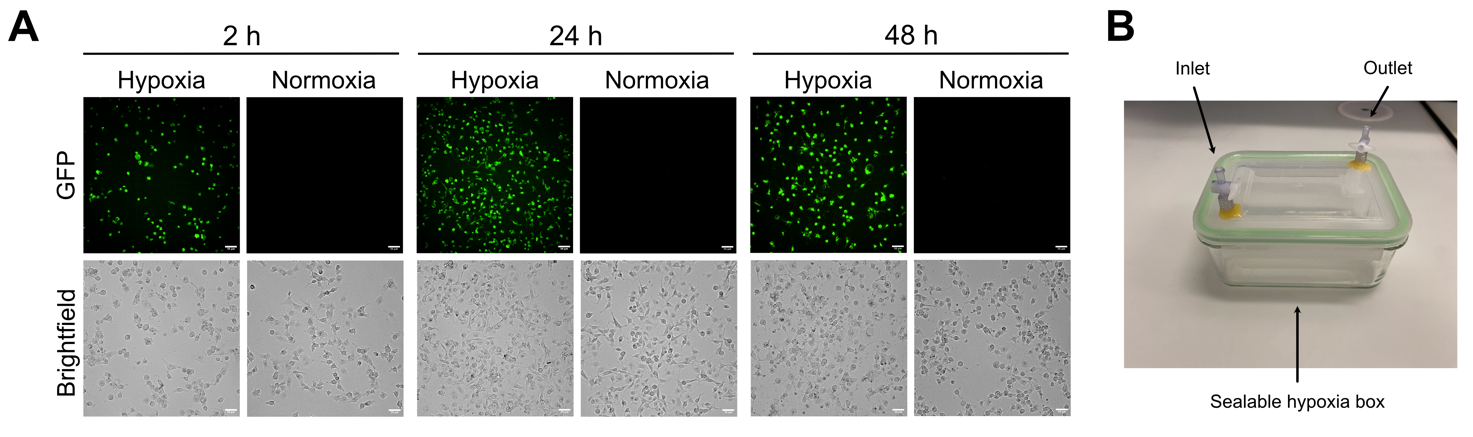


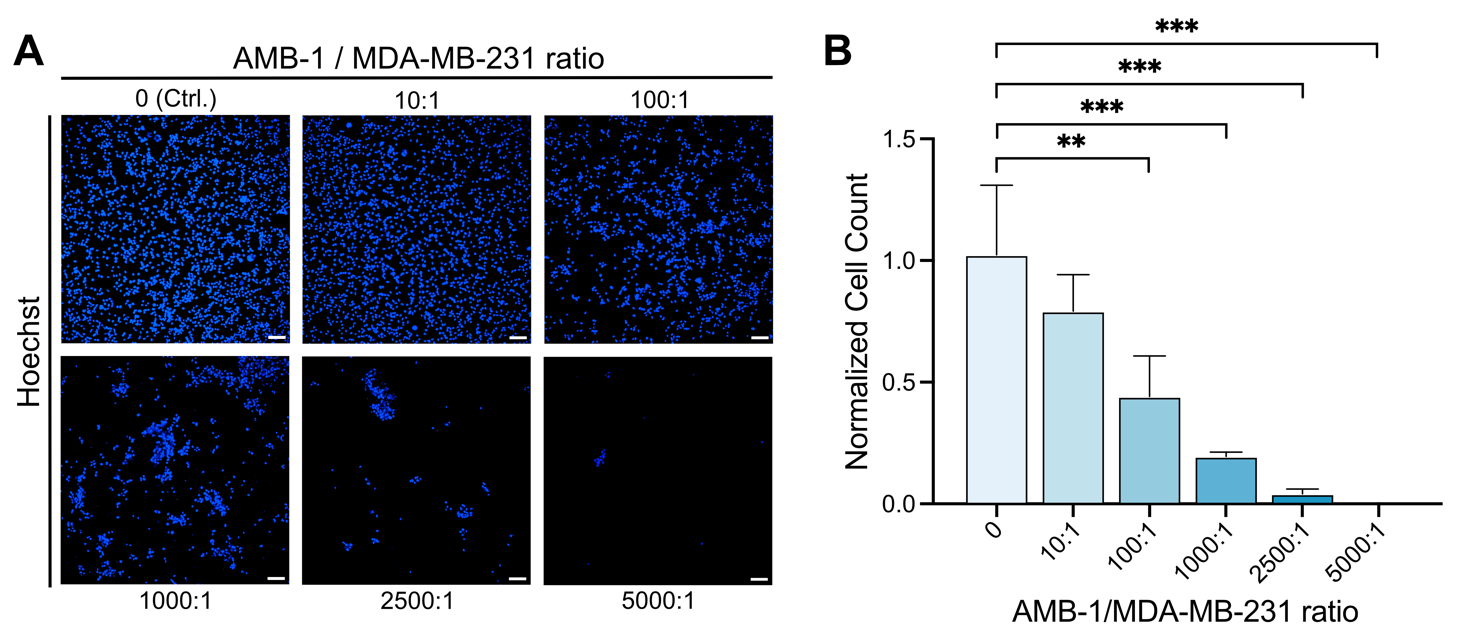


**Figure S7:** Quantification of adherent cells after incubation with AMB-1 **(A)** Representative images of human breast cancer cells co-cultured under normoxic conditions for 48 h with increasing ratios of AMB-1 bacteria. Images show MDA-MB-231 cells stained with Hoechst 33342 (blue), scale bar: 100 µm **(B)** Graphical representation of the fluorescence intensities measured in A. Cell counts where normalized to the control condition (n=3 biological replicates per condition, statistical significance was assessed with an ordinary one-way ANOVA test).


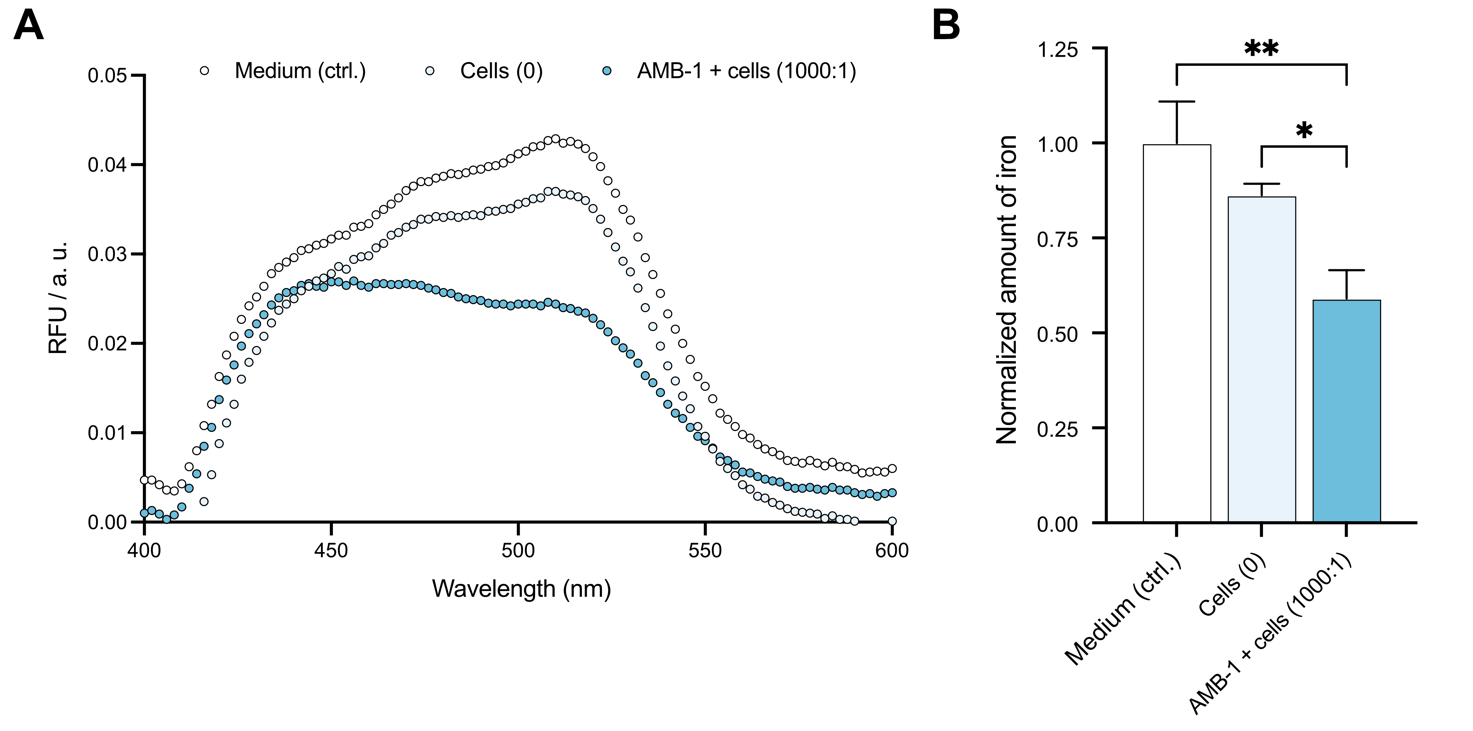


**Figure S8:** Determination of iron levels in the medium after hypoxic co-cultures **(A)** Spectroquant Iron Test was used to investigate the extent of iron consumption over 24 h. Cancer cells were either left untreated (0 Ctrl.) or incubated with AMB-1 at a bacteria to cell ratio of 1000:1. An absorbance scan was performed and the conditions were compared to the control (cell culture medium) **(B)** The intensities measured at wavelengths between 480-530 nm were averaged and the resulting mean values were normalized to the control condition and plotted as a column chart (n=3 biological replicates per condition, statistical significance was assessed with an ordinary one-way ANOVA test).


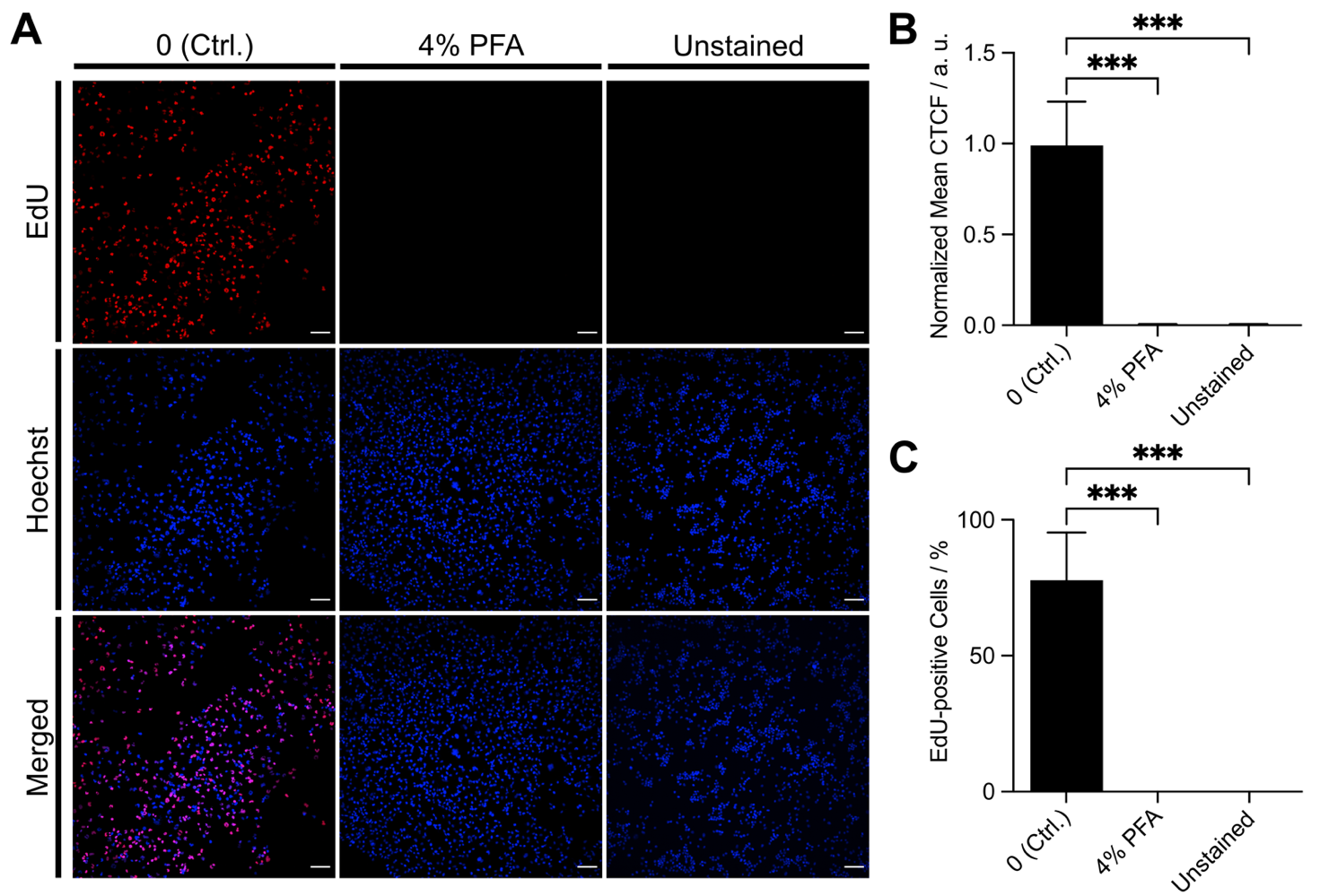


**Figure S9:** Determination of de novo DNA synthesis in MDA-MB-231 (control conditions). **(A)** EdU Kit was used to examine cellular proliferation after 24 h. Cells were either left untreated (0 Ctrl.) or treated with 4 % PFA as a negative control for cellular proliferation. Unstained cells were used as a further control. MDA-MB-231 cells were marked by EdU (red) and Hoechst 33342 (blue), scale bar: 100 µm. **(B)** The EdU fluorescence intensity of the images in Figure S2 was assessed and the normalized mean CTCF was plotted (n=3 biological replicates per condition, statistical significance was assessed with a one-way ANOVA test). **(C)** The extent of EdU-positive cells was determined by dividing EdU-stained cells by the number of Hoechst 33342 stained cells (n=3 biological replicates per condition, statistical significance was assessed with an ordinary one-way ANOVA test).


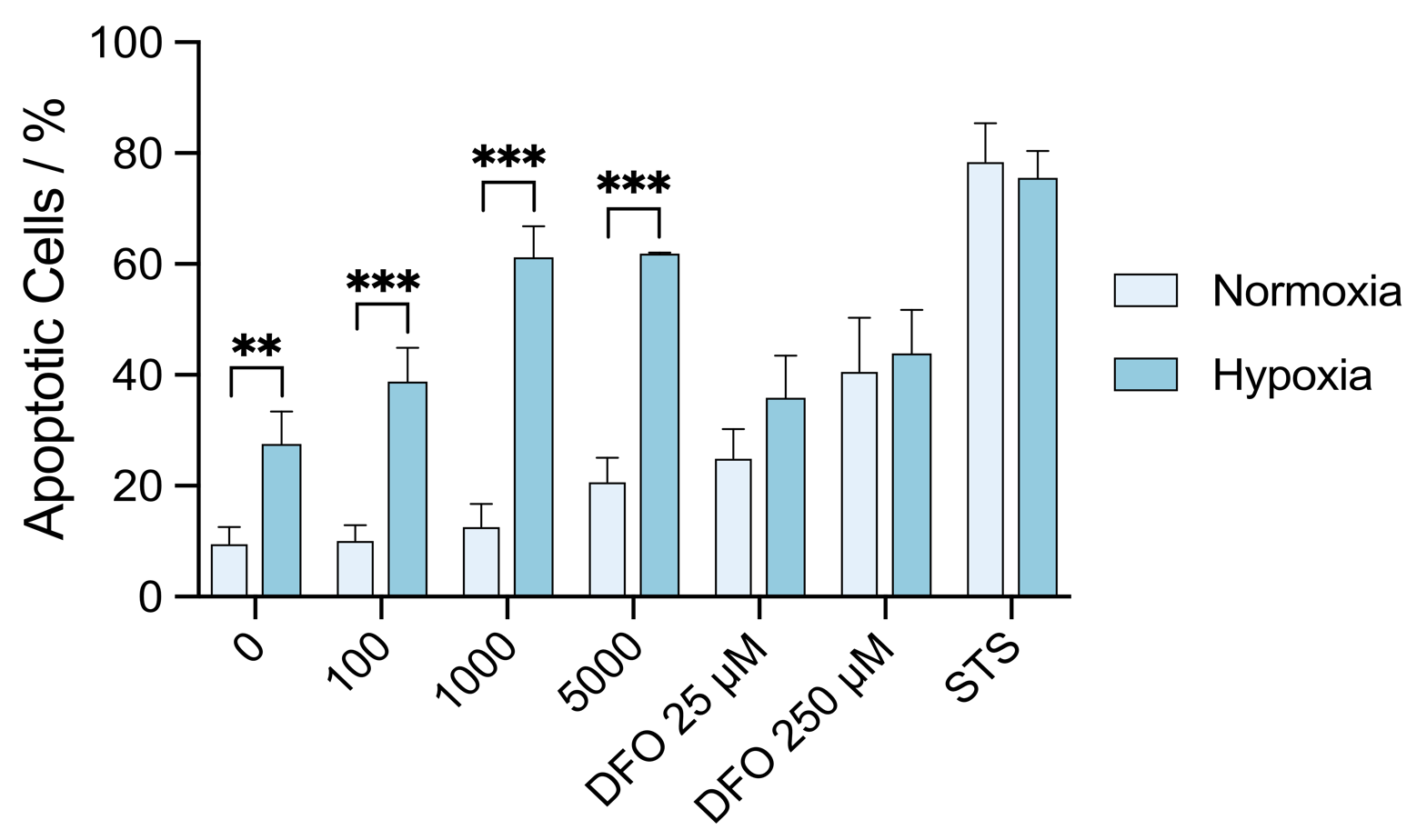


**Figure S10:** Quantification of early and late apoptotic cell populations of breast cancer cells incubated with several conditions in either a hypoxic or normoxic environment. n=3 biological replicates per condition (n=2 for STS), statistical significance was assessed with an ordinary one-way ANOVA test.


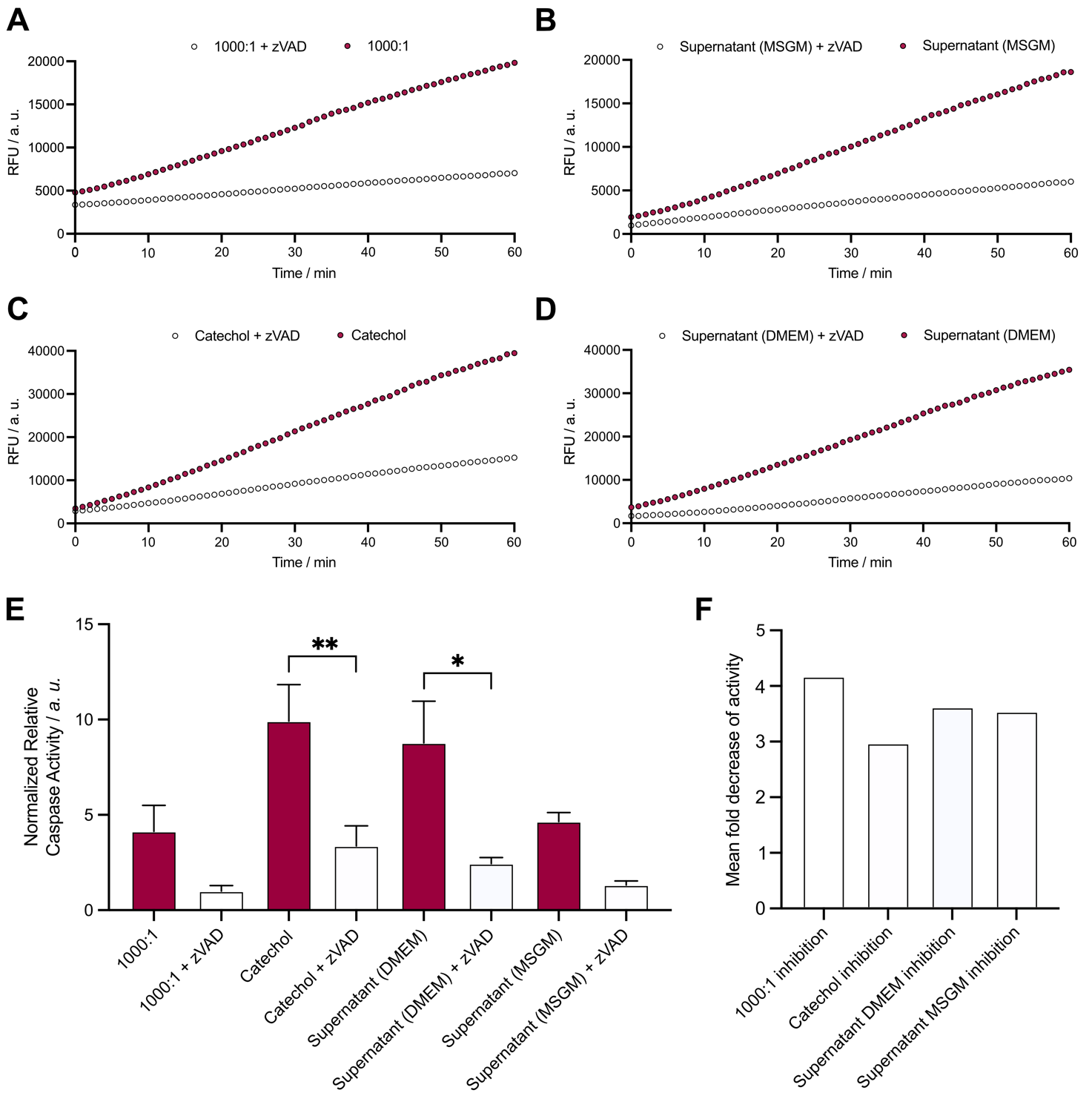


**Figure S11:** Measurement of caspase activity over 60 minutes. Mean activity of executioner caspases, with and without inhibition using zVAD, was measured (n=2 biological replicates per condition). The curves display the impact on MDA-MB-231 cells caused by (A) AMB-1 bacteria (1000:1), (B) Supernatant of bacteria cultured in DMEM, (C) Supernatant of bacteria cultured in MSGM, and (D) Catechol siderophores (1µM). (E) Relative change of caspase activity over time extrapolated from graphs A, B, C, and D was normalized to the condition with lowest activity change (1000:1 + zVAD). (F) Inhibitory effect of zVAD for each condition represented as the mean fold decrease of activity (n=2 biological replicates per condition, statistical significance was assessed with an ordinary one-way ANOVA test).

**Table 1:** List of antibodies used for Western Blotting.

| **Cat.no.** | **Product description** | **Supplier** |
| --- | --- | --- |
| AF-605-NA | Human/Mouse Caspase-3 Antibody | R&D Systems |
| AF-600-SP | Human/Mouse PARP Antibody | R&D Systems |
| AF4000-SP | Human/Mouse/Rat Actin Antibody | R&D Systems |
| A8919 | Anti-Goat IgC (whole molecule)-Peroxidase antibody | Sigma-Aldrich |
| HAF016 | Sheep IgG HRP-conjugated Antibody | R&D Systems |
