# Supplementary material for: Magnetospirillum magneticum triggers apoptotic pathways in human breast cancer cells
Source: Cancer Metab. 2023 Aug 9;11:12. doi: 10.1186/s40170-023-00313-3 (PMC10410830; doi:10.1186/s40170-023-00313-3)
Supplement: Supplementary file 2 — Additional file 2: Supplementary file 2. Fig. S12. Western blot analysis of breast cancer cell lysates. MDA-MB-231 were treated for 24 h in a hypoxic environment with either AMB-1, DFO, STS or, zVAD. Full membranes scans of PARP (A), Caspase 3 (B), and Actin (C) are hereby presented. Marked in red are lanes belonging to additional conditions that were not further investigated. [file 40170_2023_313_MOESM2_ESM.docx]

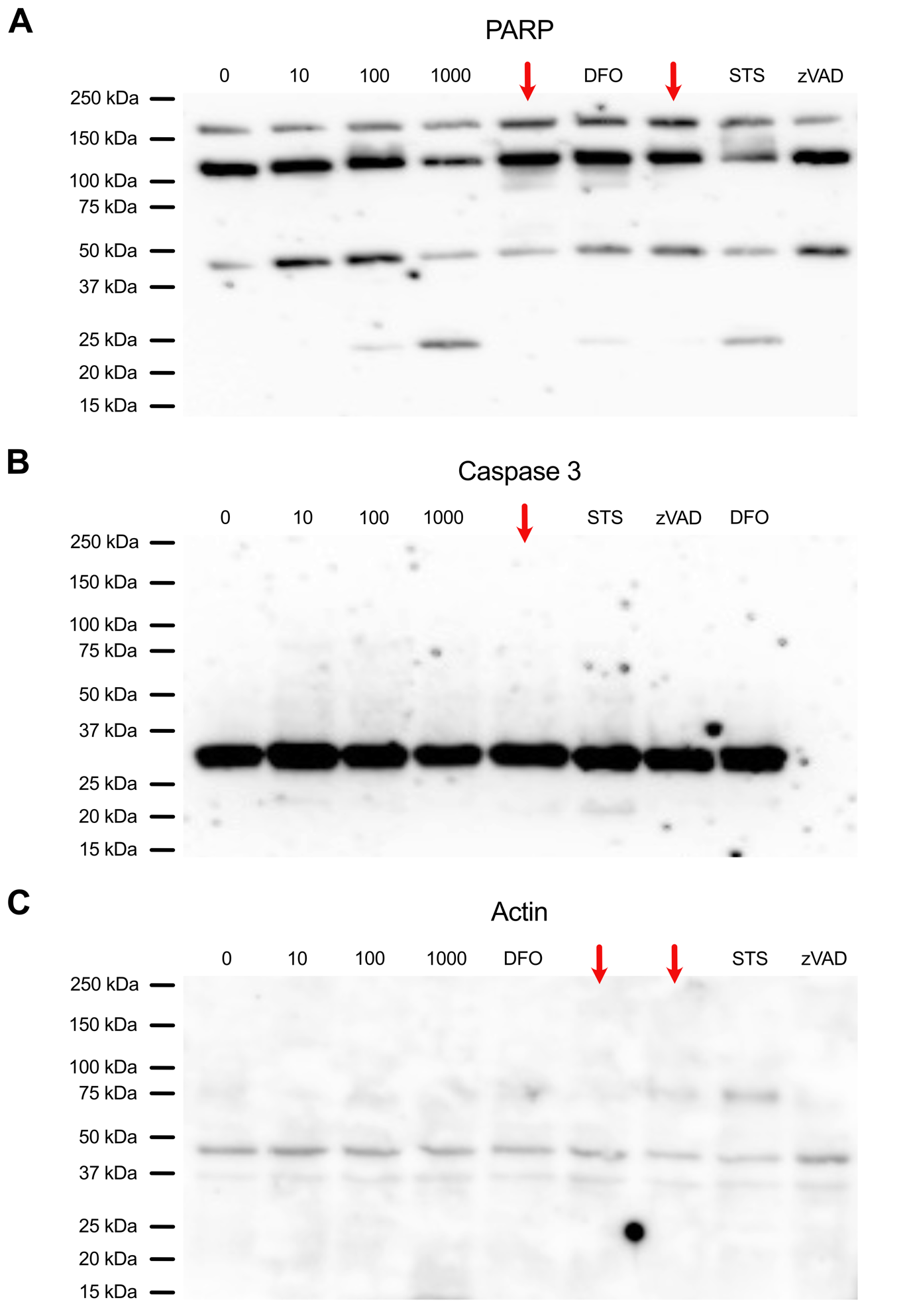


**Figure S12:** Western blot analysis of breast cancer cell lysates. MDA-MB-231 were treated for 24h in a hypoxic environment with either AMB-1, DFO, STS or, zVAD. Full membranes scans of PARP **(A)**, Caspase 3 **(B)**, and Actin (**C)** are hereby presented. Marked in red are lanes belonging to additional conditions that were not further investigated.
